# The design, launch and assessment of a new volunteer-based plant monitoring scheme for the United Kingdom

**DOI:** 10.1371/journal.pone.0215891

**Published:** 2019-04-26

**Authors:** Oliver L. Pescott, Kevin J. Walker, Felicity Harris, Hayley New, Christine M. Cheffings, Niki Newton, Mark Jitlal, John Redhead, Simon M. Smart, David B. Roy

**Affiliations:** 1 NERC Centre for Ecology and Hydrology, Crowmarsh Gifford, Wallingford, Oxfordshire, United Kingdom; 2 Botanical Society of Britain and Ireland, Harrogate, United Kingdom; 3 Plantlife, Salisbury, Wiltshire, United Kingdom; 4 Joint Nature Conservation Committee, Peterborough, United Kingdom; 5 NERC Centre for Ecology and Hydrology, Lancaster Environment Centre, Bailrigg, United Kingdom; Fred Hutchinson Cancer Research Center, UNITED STATES

## Abstract

Volunteer-based plant monitoring in the UK has focused mainly on distribution mapping; there has been less emphasis on the collection of data on plant communities and habitats. Abundance data provide different insights into ecological pattern and allow for more powerful inference when considering environmental change. Abundance monitoring for other groups of organisms is well-established in the UK, e.g. for birds and butterflies, and conservation agencies have long desired comparable schemes for plants. We describe a new citizen science scheme for the UK (the ‘National Plant Monitoring Scheme’; NPMS), with the primary aim of monitoring the abundance of plants at small scales. Scheme development emphasised volunteer flexibility through scheme co-creation and feedback, whilst retaining a rigorous approach to design. Sampling frameworks, target habitats and species, field methods and power are all described. We also evaluate several outcomes of the scheme design process, including: (i) landscape-context bias in the first two years of the scheme; (ii) the ability of different sets of indicator species to capture the main ecological gradients of UK vegetation; and, (iii) species richness bias in returns relative to a professional survey. Survey rates have been promising (over 60% of squares released have been surveyed), although upland squares are under-represented. Ecological gradients present in an ordination of an independent, unbiased, national survey were well-represented by NPMS indicator species, although further filtering to an entry-level set of easily identifiable species degraded signal in an ordination axis representing succession and disturbance. Comparison with another professional survey indicated that different biases might be present at different levels of participation within the scheme. Understanding the strengths and limitations of the NPMS will guide development, increase trust in outputs, and direct efforts for maintaining volunteer interest, as well as providing a set of ideas for other countries to experiment with.

## Introduction

Vascular plants are one of the most important indicators of the health of the environment, providing vital benefits to other taxonomic groups such as pollinators, granivorous and phytophagous invertebrates, as well as numerous other ecosystem services [1]. Due to the efforts of thousands of amateur and professional plant recorders, more is known about the vascular plant flora of the United Kingdom (UK) than probably any other country. This information has come mainly from the Botanical Society of Britain and Ireland (BSBI) who collate data from ‘opportunistic’ surveys of plants made by volunteers [2]. These observations have been summarised in two national atlases which document changes in the distribution of all native and non-native species since the mid-19th Century [3,4]. This dataset currently (2018) comprises over 35 million occurrence records and these have been used extensively to analyse changes in species frequency [4–8], assess species conservation priorities [9–11], and improve our understanding of the impacts of major environmental drivers such as nitrogen deposition [12], climate change [13,14] and invasive non-native species [15].

Despite this wealth of occurrence data, the UK still lacks an unbiased assessment of trends in abundance for the majority of plants associated with semi-natural habitats that are a priority for biodiversity conservation. Structured monitoring schemes are important in delivering this goal because they reduce the spatial and temporal biases associated with observational records collected opportunistically [8,16–18] and allow changes in the abundance of key species to be tracked annually, ideally at the habitat level. There are a number of schemes that currently meet these requirements in the UK: for butterflies [19–21], birds [22,23], and bats [24], with comparable programmes in other European countries [25]. Key to the success of these structured schemes has been the ability to track changes in the abundance of species, or species groups, in relation to hypothesised drivers such as pollution, land management, weather and climate change, pests and diseases, or invasive species. The results have been widely publicised, increasing awareness of and participation in the schemes, as well as providing environmental policy-makers and practitioners with the information needed to inform decision-making, policy and land management [26] and allowing the derivation of national biodiversity indicators [27]. These long-term monitoring datasets have also been routinely used by researchers to explore both fundamental and applied ecological questions [28,29].

Although developments in statistical modelling have improved our ability to create trend estimates from opportunistic data [30,31], data linking changes in plant abundance to their presence in specific habitats, or major environmental drivers, are still in short supply across most of the UK landscape. Trends based on grid cell occurrences cannot typically be directly attributed to habitat parcels due to the fact that such cells often overlap multiple habitats, and therefore any reported changes can often only be inferred at the level of the habitat patch using published species-habitat associations e.g. [32,33]. Exceptions include the commoner species monitored since 1970 as part of the UK Countryside Survey [34] and within a national sample of tetrads (2 × 2 km grid squares) monitored in 1986–87 and 2003–04 by BSBI recorders [35,36]. However, both schemes lack suitable design features that allow for annual trend estimates for habitats equivalent to those that are routinely being produced for birds etc. Finally, trend estimates based on grid cell occurrences can fail to detect substantial declines in abundance at finer scales, and can therefore severely underestimate change [37].

The need for a structured monitoring scheme for plants that allows for the estimation of trends in the condition of UK ‘priority habitats’ [38] has been highlighted as one of the key gaps in the UK strategy for terrestrial biodiversity surveillance [39]. This led to a scoping study to design an ‘ideal scheme’ that could: (a) detect changes in the quality of semi-natural habitats as measured by the cover of indicator plant species; (b) assist in the understanding of why these changes are occurring; (c) contribute data to relevant UK biodiversity indicators that measure change in plant communities in the wider countryside; and, (d) provide a simple, repeatable and enjoyable method that is attractive to volunteers of different levels of botanical expertise [40]. The main findings of this study were then tested and further refined over three years using a combination of expert consultation, workshops, field testing, and feedback from volunteers. This culminated in the launch of the National Plant Monitoring Scheme (NPMS) in March 2015 [41]. In this paper we describe the main design features of this new scheme; its volunteer ‘co-created’ element; and present summaries of data collected in its first two years (2015–16). We also briefly overview our methods of data capture, verification, and dissemination. The key questions that we addressed during scheme design, and, for (e), beyond, were: (a) which sampling framework to adopt; (b) which habitats and species to sample; (c) which field methods to use; (d) how to maximise scheme power; and, (e) how to maximise volunteer participation. Finally, we also investigate the potential for, and possible impacts of, various biases in the new NPMS dataset, including: an assessment of environmental and habitat biases in the monads surveyed and an investigation of human population density as the driver of these; the potential effectiveness of our indicator species for capturing environmental change; and, an assessment of the potential for biases in recorded species richnesses by surveyors.

## Methods

The core aim of the NPMS is to sample plant communities within habitats of conservation value using small plots. In what follows, the larger scale sampling framework within which these plots are located is described first, proceeding to the component parts of the method (habitats and species chosen, plot selection methodology etc.), power, pre-launch field trials and consultations, data capture, and initial investigations into data collected in the first two years (2015–16). Nomenclature for vascular plants follows the *New Flora of the British Isles*, 3^rd^ ed. [42]. Volunteers seek permission before surveying on private land, as per survey protocols. Species are not sampled during this study, only attributes (e.g. plant cover) are recorded by volunteers.

### Sampling framework and assessments of volunteer square uptake bias

One × one km squares (‘monads’) of the British and Irish grids were chosen as the basic sampling unit for the NPMS because they are feasible to survey in a day but have sufficient species diversity to maintain the interest of volunteers and provide meaningful measures of change at the landscape scale [40]. Because the NPMS is primarily focused on changes within semi-natural habitats [39], we employed a weighted-random [43] monad selection procedure based on the number and coverage of 18 UK Land Cover Map (LCM) [44] habitats of interest within each monad (excluding the littoral sediment, littoral rock, salt water, suburban and urban LCM habitats). Weights were designed to introduce a known bias towards monads with greater areas of nationally rarer habitats. For each LCM habitat the proportion *p*_*i*_ of UK monads containing any single habitat *i* is calculated as: $p_{i}=\frac{h_{i}}{\sum_{i=1}^{H}h_{i}}$, where *h*_*i*_ is the number of monads containing habitat *i*, and *H* equals 18, the number of LCM habitats of interest to the scheme. Habitat weights were then calculated as the inverse of this proportion, i.e. *w*_*i*_ = 1/*p*_*i*_, and a score *s* for each monad *j* was calculated as $s_{j}=\sum_{i=1}^{N}\frac{a_{ij}}{100}\times w_{i}$, the product of the area *a* of habitat *i* multiplied by its weight, for each of *N* habitats present in a monad. The selection probability *k* for each monad *j* was then the monad score *s*_*j*_ divided by the sum of all *J* UK monad scores: $k_{j}=s_{j}/\sum_{j=1}^{J}s_{j}$. Random numbers between 0 and 1 were then generated and matched with their corresponding monad selection probabilities. Monads with larger selection probabilities, those with larger areas of rarer habitats, were more likely to be chosen from this weighted random draw. The outcome of this process was an ordered list from which monads are to be released for survey. In order to ensure even geographic coverage at larger scales, this release process is stratified by 100 × 100 km square (hereafter termed ‘regions’), with regions containing only a few monads incorporated into adjacent regions such that the combined areas always contained more than 1,000 km^2^ of land. This process resulted in 45 regions. For the NPMS we estimated, based on other volunteer-based structured biodiversity monitoring in the UK, that 2,000 monads could potentially be surveyed annually. This number was divided between the 45 regions based on their areas, giving a range of between 11 and 77 monads per region. If more monads are subsequently required for a region then more will be released from the ordered region lists described above.

The potential for survey bias across the monads taken up by surveyors was subsequently investigated in three ways. First, by investigating the influence of human population density (variable chosen *a priori*) on the proportions of monads both allocated and surveyed within regions (analysed using binomial generalised additive models); second, by investigating the distributions of habitats surveyed by volunteers within monads relative to those habitats predicted to be present in available land-cover mapping; and third, by comparing all released, volunteer-allocated, and volunteer-surveyed monads (as of February 2017) according to a set of key environmental variables matched at the 10 x 10 km scale (variables and sources are listed in the S1 Table). The relative placement of these three sets of monads in environmental space was assessed using Principle Components Analysis.

### Habitat selection

The NPMS covers the major freshwater and terrestrial semi-natural habitats that occur in the UK (Table 1), these were based on priority habitats previously identified as being the most threatened and as requiring conservation action [45]. The only exceptions were the inclusion of montane grassland and wet heath which have no equivalent priority habitats, and the exclusion of habitats that are intensively managed or entirely anthropogenic (brownfield, improved grasslands, orchards, etc.), inherently dynamic (intertidal mud and sand flats, strandlines, mobile sand-dunes, water-bodies with fluctuating water-levels) or difficult or dangerous to survey (cliffs, open water). Some priority habitats were aggregated in order to simplify their identification in the field (e.g. dry deciduous woodland, heathland, marsh and fen, neutral grassland, rocks and scree) giving a total of 28 habitats, hereafter termed ‘fine-scale’ habitats and detailed in Table 1. At this fine scale, the distinction between ‘lowland’ and ‘upland’ types for grassland, heathland and inland rock was avoided, as it can be difficult to apply in the field; instead, we only distinguish montane types (i.e. those that occur above 600 m) from those occurring at lower altitudes [46,47]. For standing waters we avoided difficulties in differentiating mesotrophic from eutrophic types by combining them within a nutrient-rich category, as distinct from those that are dystrophic and oligotrophic (nutrient-poor). These therefore relate directly to the standard classification of British lakes [48], differentiating types A-C (dystrophic, oligotrophic) from types D-I (mesotrophic, eutrophic). Marshes and fens are some of the most challenging habitats to identify, even for experienced surveyors, and so we have employed the relatively simple distinction between those fed by acid or base-rich waters. The former includes a range of mire communities in valleys or on the margins of acidic water-bodies, as well as drainage features amongst acid bogs and acid grassland (e.g. soakways, rills, seepages, springs) and are largely, but not exclusively, confined to upland and montane regions [46]. Those fed by base-rich waters are more varied and include the majority of fen and fen-meadow communities found in the UK, including purple-moor grass and rush pastures, as well as montane flushes and mires associated with calcareous rocks [49].

**Table 1:** Broad and fine-scale habitats covered by the National Plant Monitoring Scheme (NPMS).

Broad-scale habitat	Fine-scale habitat	UK Priority Habitats [45]	Positive		Negative		Total
			Wf	Ind	Wf	Ind	
Broadleaved woodland	Dry deciduous woodland	Lowland beech and yew Woodland; Lowland mixed deciduous; Upland oakwood; Upland birchwood; Upland mixed ashwood	15	10	5	0	30
	Wet woodland	Wet Woodland	16	9	4	1	30
	Hedgerows of native species	Hedgerows	18	7	5	0	30
Native pinewood and juniper scrub	Native pinewood and juniper scrub	Native Pine Woodland	17	8	4	0	29
Heathland	Dry heathland	Lowland Heathland; Upland Heathland (part)	15	10	5	0	30
	Dry montane heathland	Upland Heathland (part); Mountain heaths and willow scrub	15	10	2	3	30
Lowland grassland	Dry acid grassland	Lowland dry acid grassland	15	10	4	1	30
	Dry calcareous grassland	Lowland calcareous grassland	19	6	4	1	30
	Neutral damp grassland	Lowland meadows (part); Upland hay meadows (part); Coastal floodplain and grazing marsh	16	10	4	0	30
	Neutral pastures and meadows	Lowland meadows; Upland hay meadows	15	10	5	0	30
Upland grassland	Montane acid grassland	-	17	9	1	3	30
	Montane calcareous grassland	Upland calcareous grassland	14	11	5	0	30
Rock outcrops, cliffs and screes	Inland rocks and scree	Inland rock outcrop and scree; Calaminarian grassland; Limestone pavement;	17	8	5	0	30
	Montane rocks and scree	Inland rock outcrop and scree; Mountain heaths and willow scrub; Calaminarian grassland	13	12	5	0	30
Bog and wet heath	Blanket bog	Blanket bog	12	13	5	0	30
	Raised bog	Lowland raised bog	12	13	5	0	30
	Wet heath	-	12	13	5	0	30
Marsh and fen	Acid-fens, flushes, mires and springs	Lowland fens; Upland flushes, fens and swamps	15	10	5	0	30
	Base-rich fens, flushes, mires and springs	Lowland fens; Upland flushes, fens and swamps; Purple moor grass and rush pasture	16	9	5	0	30
Freshwater	Nutrient-poor lakes and ponds	Oligotrophic and dystrophic lakes; Ponds; Reedbeds	14	11	1	4	30
	Nutrient-rich lakes and ponds	Mesotrophic lakes; Eutrophic standing waters; Ponds; Reedbeds	15	10	1	4	30
	Rivers and streams	Rivers	13	12	1	4	30
Coast	Coastal saltmarsh	Coastal saltmarsh	14	11	0	1	26
	Coastal sand dune	Coastal sand dunes	12	13	5	0	30
	Machair	Machair	20	6	4	0	30
	Coastal vegetated shingle	Coastal vegetated shingle	14	11	5	0	30
	Maritime cliff-top and slope	Maritime cliffs and slopes	16	9	5	0	30
Arable field margin	Arable field margin	Arable Field Margins	11	14	4	1	30

Broad and fine-scale NPMS habitats, along with their closest UK priority habitat matches. The numbers of indicator and wildflower species divided by positive/negative status and the overall totals associated with each fine-scale NPMS habitat are also provided. Wf = Wildflower level; Ind = Indicator level.

Feedback from volunteers during pre-launch field trials in 2014 (see below) suggested that our fine-scale habitats also needed to be aggregated into broader categories to allow less experienced botanists to identify them in the field (these aggregate categories are hereafter termed ‘broad-scale’ habitats; Table 1). Volunteers therefore have the option to identify habitats at either scale depending on their experience (species lists for fine-scale habitats were also aggregated into longer lists for broad-scale habitats; see below). The correspondences between fine-scale habitats and major UK and European vegetation classifications were established for the purposes of species selection, reporting and analyses (S1 File). These included British National Vegetation Classification (NVC) communities [32] and the EUNIS (Level 2) habitat classification scheme [50]. These were derived from habitat correspondence tables provided by the Joint Nature Conservation Committee (JNCC; http://jncc.defra.gov.uk/page-1425), with amendments undertaken by the authors to take into account differences between different classifications. Both broad- and fine-scale NPMS habitat descriptions, and their correspondences to NVC and EUNIS Level 2, were reviewed by a panel of five experts with specialised knowledge of European habitats, in particular coastal, montane, wetland (including bogs, fens and mires), and grassland. The feedback from these experts was considered alongside other feedback, and was used to finalise the habitat definitions, names, and the species selection step.

### Indicator species selection

The NPMS uses an indicator species approach [51], whereby volunteers record changes in the abundance of species selected by us as indicative of positive or negative changes in habitat condition. As our scheme seeks to be attractive to recorders at all skill levels, this requires the inclusion of some easily identifiable indicators that can be recognised by less experienced botanists. We named this level of participation the ‘wildflower level’. The wildflower level forms a subset of the total list of indicators, itself simply named the ‘indicator level’. Experienced recorders have the option to record all vascular plant species encountered; this is termed the ‘inventory level’.

#### Positive indicators

A pool of species was created for each fine-scale habitat as the list of all natives and long-established non-natives (archaeophytes) associated with a particular habitat, based on its constituent NVC communities [32]. Each species was then assigned a weighting based on: (i) how many fine-scale habitats it occurred in (species that occurred across many fine-scale habitats were down-weighted by 1/*n*, where *n* is the number of associated fine-scale habitats); and, (ii) by how characteristic a species was within a fine-scale habitat’s constituent NVC communities. This latter number was calculated as a species’ average (mean) abundance (where present) across the constituent NVC communities, multiplied by its average plot frequency (NVC synoptic tables give information on both typical abundances and frequencies across the plots used in the classification). The final weighting was created by multiplying these two values together; larger weights are therefore given to species that are more restricted in habitat breadth, and which are more frequent and locally abundant where they occur. Filters were then applied to remove: species requiring expert determination assessed using pre-existing expert assessments of species’ identifiability [52]; species that are unlikely to be encountered in the field due to their rarity (assessed using 2 × 2 km grid cell occurrence information from the BSBI Distribution Database, http://bsbidb.org.uk/, for 1987–2012); and, finally, species identified as negative indicators (see below). Thirty species were then initially chosen at random for each fine-scale habitat using the weightings described above. These 30 species were then reduced down to around 25 per fine-scale habitat (see Table 1 for the final counts) through a consensus expert review process (OLP, KJW, CC, authors), preferentially removing species (e.g. graminoids) which were likely to be found hard to identify by surveyors participating at the wildflower or indicator levels. Rejected species were replaced from a second list of randomly selected species (using the same weights as before), or, where no species remained, from the longer list of species associated with the constituent NVC communities of the fine-scale habitat. This *a posteriori* process of reviewing the random selections may have introduced bias in our species lists, and the potential impacts of this are assessed below (see “Assessing the effectiveness of NPMS species for capturing ecological gradients”). This selection process produced a total pool of 374 positive indicator taxa (including 11 that were also classed as negative indicators in some fine-scale habitats), of which 190 were classed as ‘easily identifiable’ [52] and could therefore be recorded at the wildflower level. Table 1 provides a numerical breakdown of the positive and negative indicator species across the wildflower and indicator levels. S2 Table presents the complete list of selected wildflower and indicator taxa.

#### Negative indicators

A pool of negative indicators for each fine-scale habitat was drawn from the Common Standards Monitoring guidance for UK terrestrial and freshwater habitats [53], with a small number of additions based on expert knowledge (KJW and CC, authors). Five species were selected for each fine-scale habitat, although only four species were available for four habitats, and only one species was selected for coastal saltmarsh (*Spartina anglica*). For 15 of the fine-scale habitats all five of the selected species were classed as easily identifiable [52], and could therefore be added to the wildflower level species lists (Table 1).

### Field methods

#### Plot size and shape

Fixed, permanent plots offer a reliable means of monitoring long-term vegetation change [54] and were chosen for the NPMS because they are easier to relocate, set-up and record than plots newly located at random each year, transects, or more complex plot designs, e.g. nested plots. In addition, in order to achieve a given level of power over the short- to medium-term, fewer permanent plots should be required compared to new random plots annually, due to the fact that revisiting sampling locations reduces the site variance component of an estimated trend [55]. For the NPMS, the size and the shape of the plots were determined by a review of the relevant literature [40,56], followed by pre-launch field trials and reviews with volunteers (see below); a particular emphasis was placed on comparability with the UK Countryside Survey [57] to facilitate future joint analyses and comparisons. These processes suggested a size of 25 m^2^ (5 × 5 m or linear equivalent) for open terrestrial and freshwater habitats dominated by grasses, herbs and small shrubs, whereas 100 m^2^ (10 × 10 m) was considered better suited for woodlands (Table 2). Square plots were chosen for most terrestrial habitats, with the option to use linear plots of equivalent area if a habitat patch is too narrow to accommodate a 5 × 5 m plot, for example when it is located on a road verge [58]. Linear plots (1 x 25 m) are also used for hedgerows, the margins of water bodies, inland rocks and scree, and arable field margins. Further information on these sampling approaches can be found in NPMS [58].

**Table 2:** The size and shape of plots used for surveying different habitat types within the NPMS. See [58] for more detail. S = square; L = linear.

NPMS habitat	Plot area (m^2^)	Plot shape	Plot dimensions (m)^a^	Comment
Woodland	100	S	10 × 10	-
Hedgerows	25	L	25 × 1	-
Dry heathland	25	S/L	5 × 5 or linear equivalent	-
Grassland	25	S/L	5 × 5 or linear equivalent	-
Rocks and scree	25	S/L	5 × 5 (horizontal), 12.5 × 2 (vertical)	-
Bogs and wet heath	25	S	5 × 5	Can include bog pools
Marsh and fen	25	S/L	5 × 5 or linear equivalent	Small mires/springs included if >50% of plot
Freshwater	25	L	25 × 1	From high water level
Saltmarsh, dunes, machair, shingle	25	S	5 × 5	Excludes pioneer saltmarsh and strandlines
Maritime cliffs and slopes	25	S/L	5 × 5 or linear equivalent	Excludes the cliffs themselves
Arable field margins	25	S	25 × 1	Ground flora only

^a^The linear equivalents for a 25 m^2^ plot are: 4 × 6.25 m; 3 × 8.3 m; 2 × 12.5 m; 1 × 25 m.

#### Plot placement and the minimisation of bias

**Fig 1:**
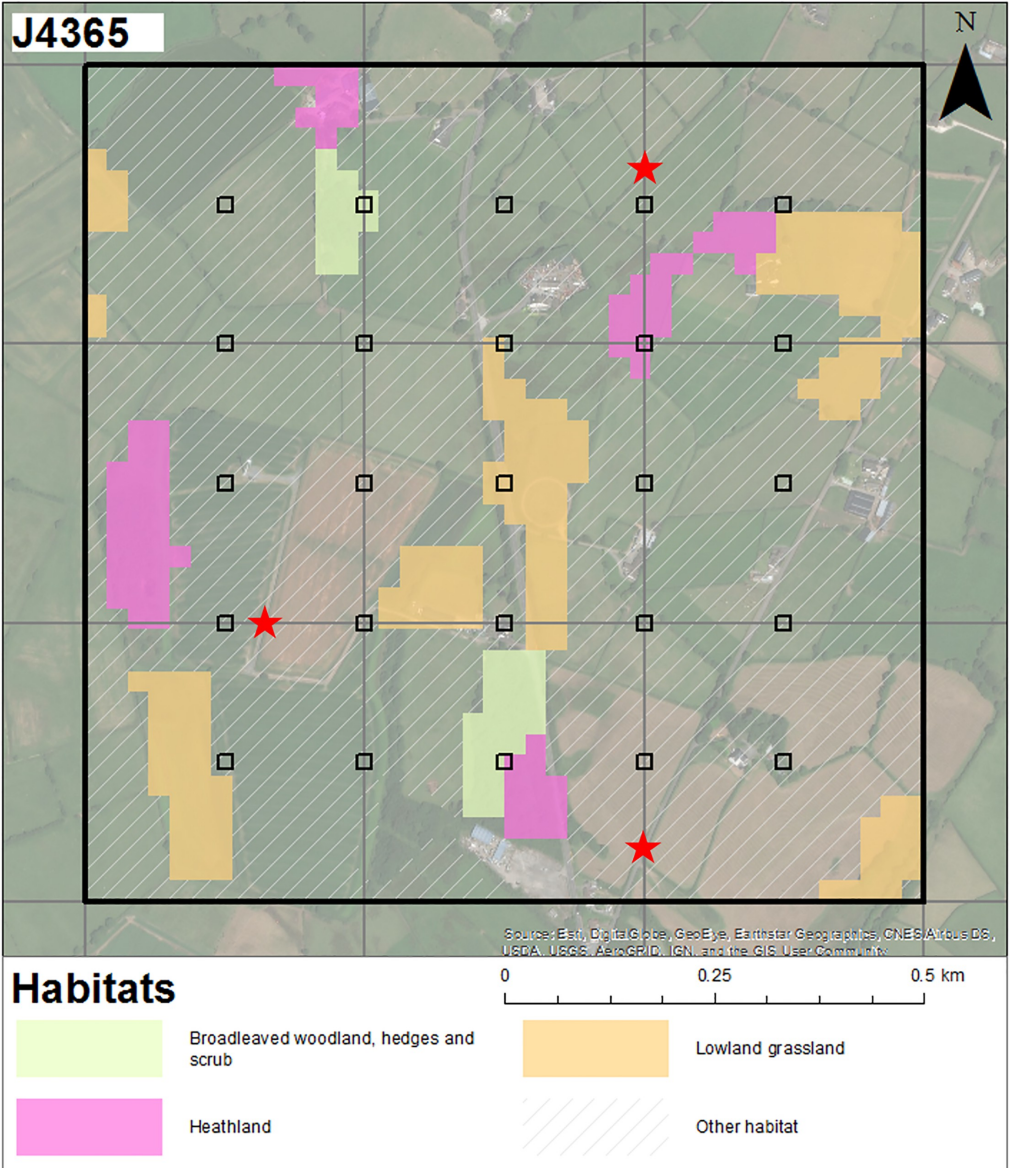
An NPMS survey square. An example of an NPMS one kilometre square (monad) showing the locations of randomly selected plot locations in relation to semi-natural habitats. These are positioned at the intersections of a fixed grid, with the exception of those plots that would intersect urban areas. The possible locations of three accessible linear plots along margins of arable fields or hedgerows are indicated with red stars. Base map attribution: Esri, DigitalGlobe, GeoEye, Earthstar Geographics, CNES/Airbus DS, USDA, USGS, AerGRID, IGN and the GIS User Community.

One of the most challenging elements of the design process was to reduce bias in plot placement; bias can significantly reduce the ability of monitoring schemes to detect representative change, especially if volunteers record in atypical areas, such as those that are the most species-rich or the most disturbed [8,59]. A set of options emerged from a two-day workshop held in February 2014, at which 16 statisticians, monitoring experts, and representatives of the NPMS partner organisations discussed all design aspects of the proposed scheme. These options were then subject to field trials in 2014 (see below). The final preferred option was to provide volunteers with up to 25 pre-selected locations in their sample monad, located at the intersections of a fixed grid (Fig 1). The use of a grid, aligned to either the British, Irish or UTM30 grid (Channel Islands), means that plots are randomly placed with respect to the land surface. We note that such ‘systematic’ sampling schemes have theoretical implications for the estimation of variance [60], although in practice such issues are often dealt with in ecology using model-based inference [55].

To assist with plot selection in the field, volunteers are provided with a map showing the potential locations of plots according to the grid-based sampling strategy, and areas of NPMS habitats predicted to occur in the sample monad (based on national inventories of semi-natural habitats and the 2007 LCM, matching the classifications used in these to NPMS habitat types; Fig 1). Volunteers visit these locations and attempt to record three square and two linear plots where these coincide with the semi-natural habitats listed in Table 1, with the aim of recording as many different habitats as possible. Square plots are recorded at intersections of the grid, as described above (numbered plots in Fig 1). Linear habitats are recorded from where these intersect grid lines (demonstrated by the red stars in Fig 1). The number of square and linear plots surveyed will vary depending on the habitats present in the monad, as well as on accessibility and surveyor safety considerations; where options for the implementation of these methods are limited, the final option of self-selecting plot locations in representative areas of habitat elsewhere in the monad exists [58]. These can include small habitats that rarely intersect grids, such as ponds and flushes. Alternatively, volunteers can record up to three plots in habitats not listed in Table 1, although these should not be recorded at the expense of the NPMS habitats; the idea here being that plots recorded in ‘out of scheme’ habitats may one day change. This strategy also serves to help minimise bias, in that the option for habitat expansion, creation, or restoration is further accounted for.

#### Recording abundance and other attributes

To increase the chances of finding all species sought, volunteers are encouraged to visit their plots twice a year, once in late spring or early summer and once in late summer [58]. Within each plot the abundance of the species found (depending on survey level) is estimated using a (quasi-)logarithmic ordinal scale of cover-abundance (the Domin scale), that emphasises proportional changes in cover [61]. This approach was chosen because it minimises subjective biases in cover estimation as it is easier, and quicker, to assign species to broad classes than to assign a percentage cover integer [62,63]. It is therefore likely to be more suitable for use by untrained volunteers [64]. In addition to this, feedback from our field workshop (see below) suggested that the use of an interval-based scale reduced volunteer anxiety around assigning precise integer cover values. The NPMS protocol also asks for the cover of bare earth, plant litter, bare rock or gravel, and bryophytes and lichens to be estimated. Other information recorded for plots includes aspect, slope, vegetation height (percentage of vegetation in five different height bands), how wooded the plot is, and the grazing intensity [58].

#### Plot re-visitation

The NPMS has been designed to provide annual trends in the abundance of key species and so volunteers are encouraged to re-visit their plots annually, or at the very least twice every five years. To aid with relocation volunteers are asked to record the position of their plots by drawing a detailed sketch map; the capture of plot coordinates using GPS is also encouraged. Volunteers are advised against permanently marking plots. A flow diagram overviewing the whole NPMS survey process can be found in Walker et al. [41] or viewed on the scheme website (www.npms.org.uk/content/resources).

### Scheme power

The power of the scheme for detecting species-level changes in abundance was assessed using two approaches: a frequentist non-hierarchical linear model for interval-censored cover data and a Bayesian hierarchical model for the same data type. Details are discussed in full elsewhere [65]. The models were applied to data simulated using a hierarchical framework [66], with site-dependent slopes and intercepts. Standard deviation hyper-parameters used in the simulation of data were estimated from UK Countryside Survey vegetation data [57]; 100 simulations were used for each scenario (i.e. a unique combination of simulated decline, starting abundance, and number of sites).

### Pre-launch field testing: The NPMS as ‘co-created’ citizen science

As described above in relation to habitat selection and field methods, various aspects of the NPMS were field tested in 2014 by a combination of BSBI plant recorders and participants in Plantlife’s ‘Wildflowers Count’ (WFC) scheme, an outreach-focused forerunner to the NPMS. Citizen science (CS) can be described as the contribution of volunteers (often amateurs, but not necessarily) to scientific research; this area of activity has been broken down into various sub-categories by different authors. For example, Pocock et al. [67] reviewed a suite of existing CS classifications, contrasting the established model of biological recording in Britain and Ireland with other systems. One oft-discussed type is ‘co-created’ or ‘participatory’ CS [67], where volunteers contribute towards the selection of objectives and/or the methods used within a study, as well as towards data collection. Some commentators suggest that this model is more empowering than what might be referred to as more traditional ‘contributory’ CS [67], in that volunteers are not merely passive recipients, but actively help to construct elements of a project [68]. Volunteer input to NPMS design was sought in three main ways: (i) through a small residential field workshop; (ii) through a distributed field trial, in which a larger, self-selected set of volunteers were randomly assigned competing field methods; and, (iii) through a web-based survey to all volunteers participating in the transitional year (2014) between the existing scheme and the new NPMS. These activities are briefly described below, along with representative volunteer feedback and the ways in which the design of the NPMS was influenced as a result.

#### Residential field workshop

Six volunteers and four representatives of NPMS partner organisations spent two days discussing and trialling competing design options, including issues of plot placement, plot size, the provision of GIS data on maps (e.g. Fig 1), habitat identification and target species encounter rate; general improvements to survey guidance were also discussed as they arose. Participants were divided into four groups such that each group contained a mix of project representatives and volunteers; on day one each group was assigned a random combination of plot selection method and GIS mapping layer (similar to Fig 1); on day two groups were assigned one of two plot sizes (5 × 5 m or 10 × 10 m) and a habitat type. Different local monads were used on each day, but all groups trialled their separate methodologies in the same monad. Plot selection methods for day one included the finally selected ‘gridded’ method (Fig 1); a completely random set of 30 plots within a monad; and, a ‘targeted’ set where five plots were placed randomly within habitat parcels identified from the GIS habitat layer being used. The choice of GIS layer was relevant for all plot selection methods, given that surveyors are encouraged to select plots in NPMS habitats, and to maximise habitat coverage. The two GIS mapping layers investigated were the 2007 LCM [44] and the Natural England Priority Habitat Inventory (https://data.gov.uk/dataset/priority-habitat-inventory-england). Discussions in the field and debriefing sessions resulted in support for the gridded plot selection method. This was due to the increased ease of navigation between plots, the even spread of plots across a monad, with plenty of choice, and the fact that, visually, grids were considered to be more ‘intuitive’ by volunteers, compared to a random distribution. The random distribution created confusion due to the fact that some volunteers considered the random examples to be too clumped (i.e. their expectation of random was closer to an over-dispersed pattern). The gridded approach is also clearly less dependent on the accuracy of the habitat mapping data used. The field trials also supported more flexibility in the assignment of plot sizes to habitats (Table 2), the creation of the additional ‘broad-scale’ level of habitat classification mentioned above, and other small additional improvements, such as the combination of some species at the wildflower and indicator levels e.g. *Viola riviniana* and *V*. *reichenbachiana* are recorded as one aggregate unit at these levels (S2 Table).

#### Volunteer field trials

Around 3500 volunteers were registered with the WFC in mid-2014. These surveyors were emailed asking for expressions of interest in trialling new survey methods within their existing WFC monads. Ninety-three surveyors responded and were assigned random combinations of the three plot selection methods and two GIS base mapping options described above, with customised maps provided; 38 of these 93 (41%) completed the trial according to their assigned methodology—drop-out was typically due to the perceived complexity of the trial or to a lack of time. Web-based evaluation questionnaires were sent out before the survey; paper copies were also provided so that volunteers could easily consider the questions whilst in the field. A summary report on the outcomes of this exercise is included in S2 File. The questionnaire was based on the web consultation (see below), but with additional questions relating to the experimental aspect of the field trial.

### Web consultation

The need to transition from an existing scheme (WFC) to a new methodology whilst retaining volunteers meant that all WFC volunteers were given the option to use an early version of the NPMS methodology in 2014, representing another learning opportunity for the design of the final scheme. Previously WFC volunteers used either a transect-based or self-selected plot approach, but with different focal habitats and indicator species. Design changes introduced in 2014 related therefore to species and habitat identification, as well as to the plot selection protocol. All existing volunteers were given the choice between carrying on with their WFC methodology and recording new plots based around the habitat and species lists formulated at that point in the scheme’s development. The web-based consultation was sent out before the end of the field season; phone-calls, email enquiries, and feedback received during training sessions were also logged; the full questionnaire is available in S3 File.

### Data capture, verification and dissemination

NPMS data are captured through a website (www.npms.org.uk) based on the open-source Indicia toolkit (www.indicia.org.uk). The data entry process is supported by online guidance documents, YouTube videos, data entry workshops and the project coordinator; an app is also available. The foundations of the NPMS website on the Indicia toolkit enables the scheme to take advantage of pre-existing web infrastructure for general biological recording in Britain and Ireland (www.brc.ac.uk/irecord), including infrastructure for community record verification and wider data dissemination [69]. Records of species that are classed as easy to identify according to the criteria mentioned above [52] that are within their known hectad range are automatically accepted (although can be reviewed), whilst all other records are flagged as requiring verification by a local expert. Annual datasets, including record-level verification statuses, are shared with the UK National Biodiversity Network (www.nbn.org.uk; a GBIF node) and deposited with the NERC CEH Environmental Information Data Centre (http://eidc.ceh.ac.uk).

### Assessing the effectiveness of NPMS species for capturing ecological gradients

Our selection of indicator species sought to meet various requirements, with semi-natural habitat associations and local abundance being important considerations. Whether these indicator choices will ultimately be able to assist in the identification of drivers of environmental change through the representation of ecological gradients is an important question. The existence of unbiased samples of British plant communities in the form of the 2007 Countryside Survey [57] allows us to investigate this. A subset of quadrat data from the Countryside Survey was selected to represent the plot sizes and habitat types covered by the NPMS. Detrended Correspondence Analysis (DCA) was used to extract the main ecological gradients from this dataset. The first two DCA axes were interpreted as substrate fertility and succession/disturbance [70]. The Countryside Survey quadrat data were then filtered to NPMS indicator and wildflower species, with new scores subsequently calculated for the filtered plots for DCA axes 1 and 2. Filtered plot scores were then compared to their paired ‘full’ plot scores to examine correspondence between the full ordination and the reduced plots.

### Assessing potential recording biases

Despite the existence of protocols and other guidance, all surveys have the potential for issues with species’ detectability or other biases, but this may be a particular issue with volunteer-based surveys [71–73]. The existence of a separate survey contemporary to the NPMS covering an overlapping set of habitats in Wales, namely the Glastir Monitoring and Evaluation Program (GMEP; www.gmep.wales), allows for a test of the potential for this to have affected NPMS data. The GMEP survey follows the methodology for unbiased plot placement used in the Countryside Survey [57], with recording performed by contracted surveyors. GMEP data for 2014 and 2016 were compared to NPMS data (2015–16) on the following basis: plots were matched on broad habitats [74] and were limited to comparable plot sizes. The distribution of NPMS monad weights (see above) for the two sets of monads were similar, suggesting that they were similar samples of the landscape, and reducing the chance that detected differences were solely due to the sets of monads visited. GMEP and NPMS inventory level plot data were then filtered to retain only NPMS indicator species; this was in order that all comparisons were based on the same set of species. Indicator richness was calculated as the sum of all unique indicator species recorded within a plot across the two years of each monitoring program, thus removing issues relating to the non-independence of re-surveys of a single plot. Indicator richness data were then modelled as a function of broad habitat and survey type, and their interaction, in Poisson GLMMs with a random intercept for each monad to account for spatial autocorrelation at local scales. Separate models were run using the lme4 package in R [75] for GMEP vs NPMS indicator plots and for GMEP vs NPMS inventory plots. *Post hoc* comparisons were carried out using the lsmeans package in R [76] to investigate the deviation of the NPMS plot type from GMEP plots within a broad habitat. The presence of remaining spatial autocorrelation in the model residuals was assessed using Moran’s *I*.

## Results

### Scheme uptake

**Fig 2:**
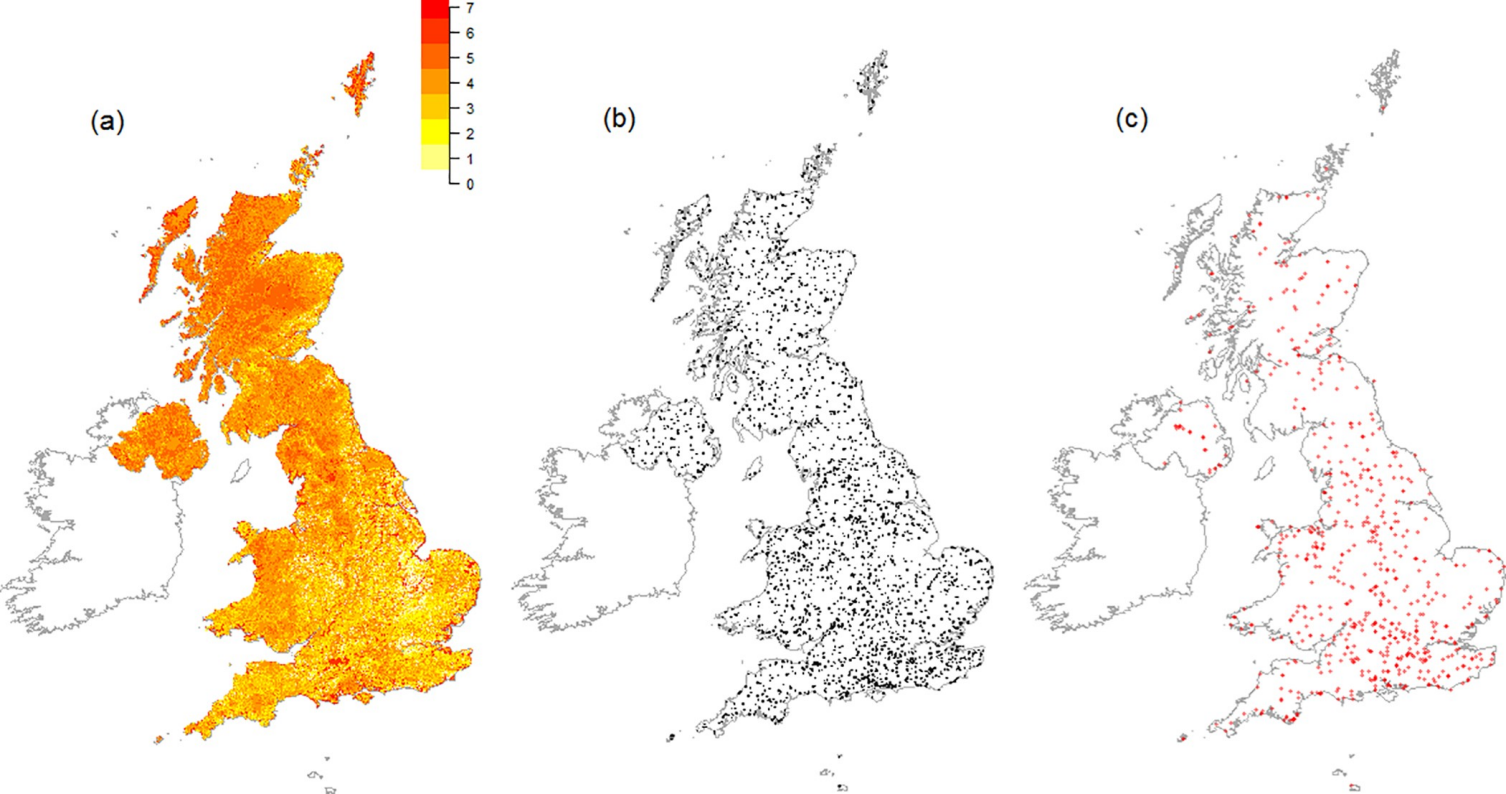
Land cover weightings, released monads, and surveyed monads to 2016. (a) The distribution of monad weights across Britain and Northern Ireland (weights were natural log transformed for visualisation, the colour gradient legend gives these transformed weights); (b) the monads that have been released for survey up to the end of 2016; (c) monads surveyed by volunteers in 2015 and 2016.

**Fig 3:**
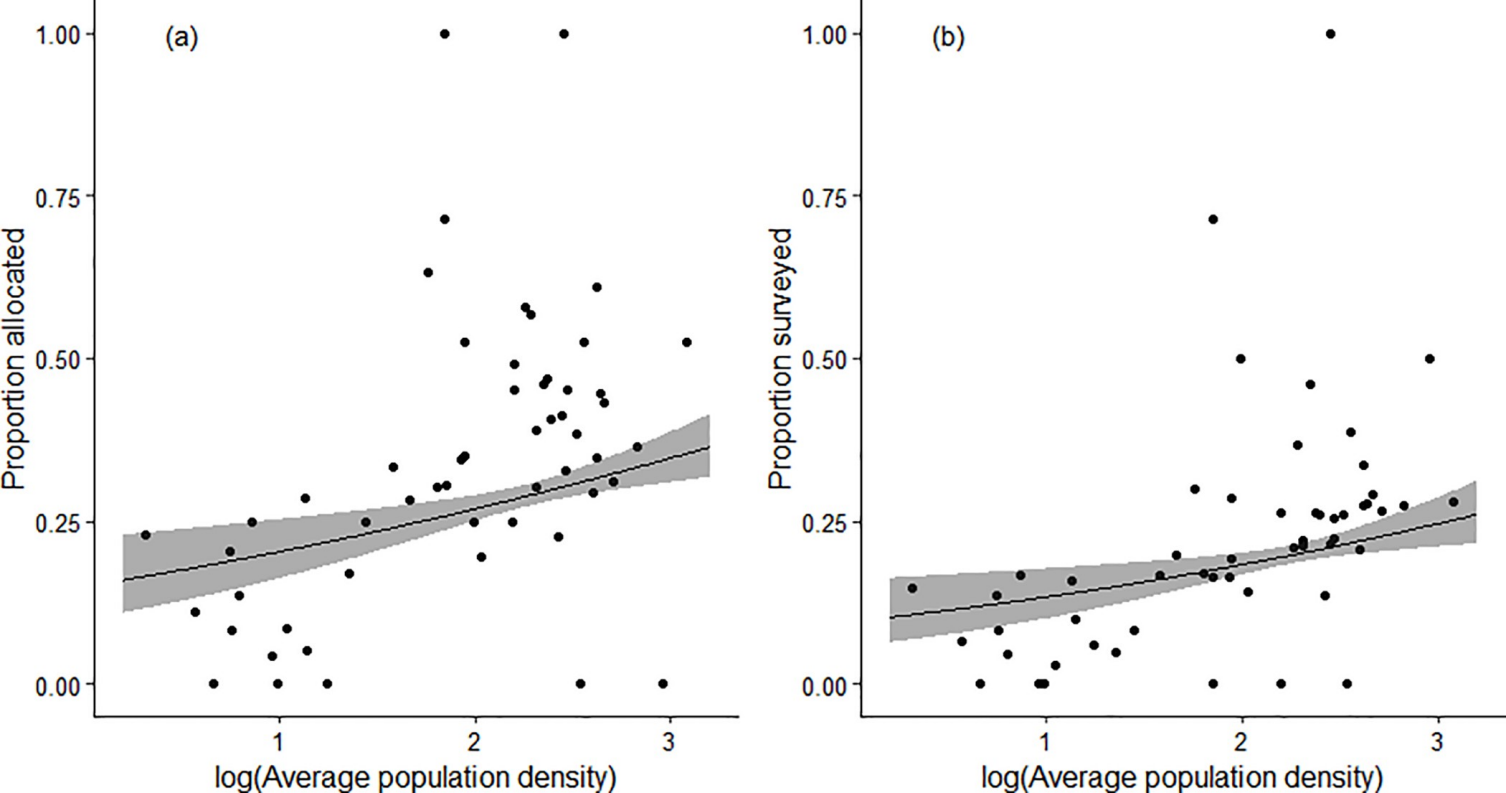
The relationships between monad allocation/survey status and population density. (a) The relationship between the proportion of allocated monads per 100 x 100 km region as a function of population density; (b) The relationship between the proportion of surveyed monads per 100 x 100 km region as a function of population density. Grey ribbons represent 95% confidence intervals.

Fig 2C maps the monads for which data were received during the first two years of the scheme. Two thousand six hundred and eighty-seven monads had been released as of February 2017 (Fig 2B), 18 of these were removed for reasons of land access. Of these, 1039 had been allocated to volunteers (38.9%), with data received for 642 (Fig 2C; 61.8% of those released, and 24.1% of those available for survey). Within 100 × 100 km areas of the British grid (‘regions’), including those extended to cover Northern Ireland, a binomial generalised additive model of the effect of average human population density in the year 2000 [77] on the proportion of monads allocated out of the total number released was significant (*z* = 3.44, *P* < 0.001; no overdispersion detected); this model included a smooth term on latitude and longitude to account for spatial autocorrelation, which was found to be present in a model without a spatial term. The fitted model indicated that a change in average population density from 10 to 100 persons km^-2^ within a region was related to a 6.3 percentage point increase in the proportion of monads allocated (Fig 3A). A similar model for the proportion of monads surveyed (as opposed to just allocated), again accounting for spatial autocorrelation, gave a comparable result (*z* = 2.93, *P* = 0.003; no overdispersion detected; Fig 3B), with the same change in population density giving a 4.7 percentage point increase in the proportion of monads surveyed.

### Volunteer field trials and web consultation

The self-selected pool of volunteer field testers were characterised by significant field experience (80% with >3 years survey experience), whereas the more general web consultation (described below) was intended to capture a broader cross-section of the volunteer community. A number of recommendations emerged from the field trials, and a selection of volunteer comments are provided in the S4 File. The recommendations could be broadly divided into those that were issues of survey flexibility and those that were issues of communication or guidance, although this division was not absolute. Issues of flexibility included: (i) the need for a clear protocol for plot self-selection when the systematic approach (Fig 1) fails to provide sufficient locations within a monad, for example due to land accessibility issues; (ii) allowing for variation in the number of square and linear plots surveyed; (iii) an acknowledgment that plot sizes should vary by habitat (as found for the residential field workshop); (iv) allowing surveyors to survey different habitats at different survey levels (i.e. wildflower, indicator and inventory) within a monad; (v) adjustments to the species selection method to increase species encounter rate (reflected in the final method described in the ‘Indicator species selection’ section above).

Issues of communication or guidance included: (i) encouragement to volunteers to attempt to distribute plots across different habitat types; (ii) explaining that although the indicator level includes almost 400 species (S2 Table), the within-monad habitat focus restricts the number encountered considerably; (iii) ensuring that the meaning of the inventory level was understood (i.e. record all species, not just all species that a surveyor can identify); (iv) improvements to online data entry and guidance; (v) varying GIS base mapping by the best available dataset for a region, and reclassifying mapped habitats to the NPMS definitions to provide more support for habitat identification in the field; (vi) ensuring time commitments are clearly communicated, particularly that they will reduce through time after plots are initially identified in year one [41]; and, (vii), clearly communicating the aims of the survey in order to mitigate against low interest due to species-poor plots at the wildflower and indicator levels. These opportunities to learn from our volunteers directly influenced the choice of methods described earlier in this paper.

Three-hundred and sixty-six responses were received for the web consultation, around 10% of the volunteer base at that time; however, the majority (74%) of these respondents had opted to continue with the old methodology, leaving 26% of respondents commenting on aspects of NPMS design. Many of the same issues arose as from the structured field trials described above, i.e. evidence that the methods should include elements of flexibility in order to reduce volunteer attrition, and an emphasis on communication, guidance and support. Again, representative volunteer feedback on several topics is given in the S4 File to demonstrate the range of feeling and engagement encountered. The expertise and understanding of numerous surveyors is particularly evident, as is the fact that surveyors typically demonstrated a strong desire to get things right. It is clear that a good proportion of surveyors took advantage of the opportunity to negotiate the methodology of the new scheme.

### Levels of participation

**Fig 4:**
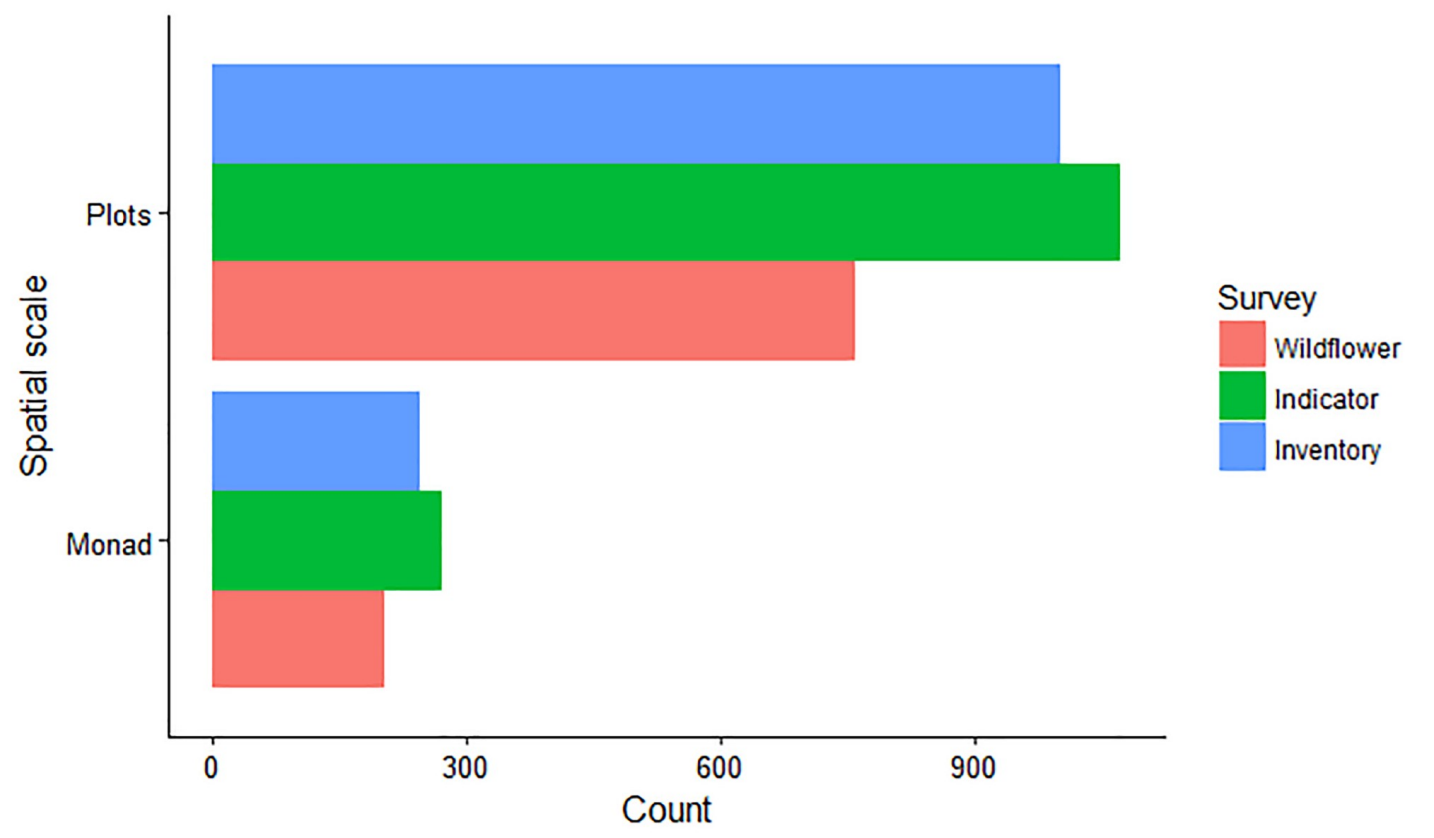
Counts of surveyed monads and plots per participation level. The distribution of effort (2015–16) across survey levels at two different spatial scales.

NPMS surveyors may participate at one of three levels, where the levels are linked to the number of species searched for per habitat (Table 1). At the level of the monad within which surveyors set up their habitat plots (see Fig 1), the distribution of participants’ chosen survey levels was relatively evenly distributed (Fig 4). However, at the level of the individual habitat plot, there was a clear tendency for volunteers choosing the intermediate and higher level of the scheme to record more plots (Fig 4). The modal number of plots per monad in both years of the survey was five. This was the commonest number of plots relative to the next highest category (four plots per monad) by a factor of almost three in 2016, suggesting that participants were trying to follow the recommendation in the NPMS guidance booklet to record five plots [58].

### Habitat coverage

**Fig 5:**
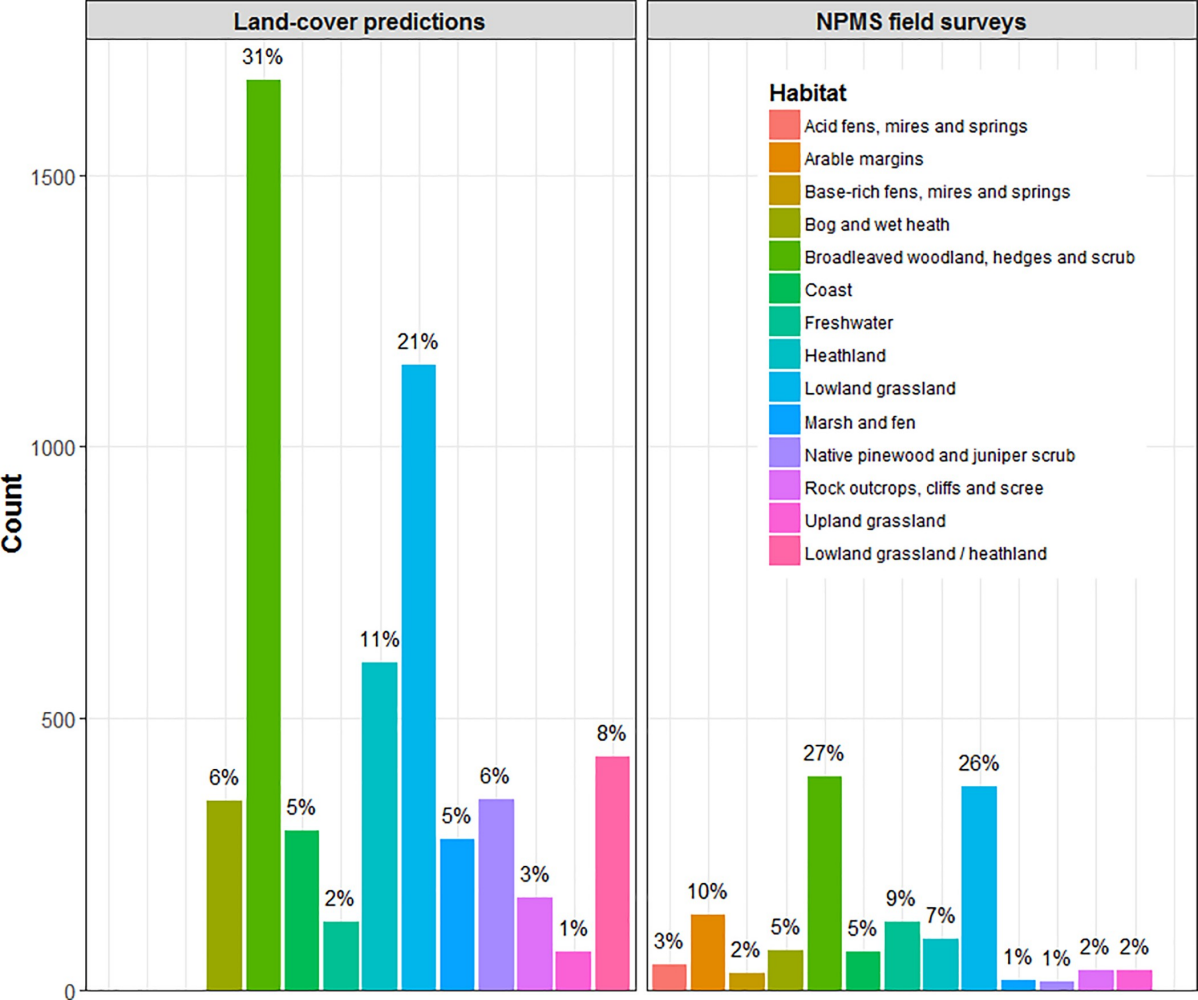
Land cover inventory information per monad compared to NPMS surveyor returns. The occurrence of NPMS broad habitat types across the monads released for survey (2015–16; *n* = 2687). Frequencies of land cover types from existing inventories are compared with the frequency with which broad habitats were found at least once within a monad by volunteers (percentages are also given for easier comparison of the relative composition of the two information types). Land cover data comes from the LCM 2007, the Natural England Priority Habitats Inventory, and the Welsh Phase One map, with categories matched to NPMS broad habitats.

The weighted-random selection of monads for release resulted in an intentional bias towards those monads with larger areas of nationally rarer habitats (Fig 2A). The heat map of monad weights (Fig 2A) indicates that this strategy led to higher selection probabilities for coastal and freshwater wetland habitats; other notable hotspots included the large area of unimproved calcareous grassland on Salisbury Plain. Despite the selection methodology underlying these weightings, and the monad release and habitat plot selection strategies, surveyor activities in the field will be influenced by other factors, including land access and habitat or species identification confidence. Fig 5 compares information on NPMS broad habitat types derived from land-cover mapping products with the frequency with which broad habitats were reported at least once from within a monad by volunteers. Land-cover product-derived habitat information was predicted by matching habitat types from three land cover inventories (LCM 2007 [44], the Natural England Priority Habitats Inventory, and the Welsh Phase One map [78]) with NPMS broad habitat categories.

Broadleaved woodland and lowland grassland are widely surveyed, as expected from mapping data, whereas other habitat types, e.g. acid grassland/heathland and montane grassland, are likely to be under-represented in relation to these GIS data (Fig 5). Coastal habitats may be over-represented. There is also good take-up of habitats that are not represented by the land-cover products, namely arable field margins, and small acidic or basic wetland features (i.e. fens, mires, and springs).

### How well does the NPMS sample UK environmental space?

**Fig 6:**
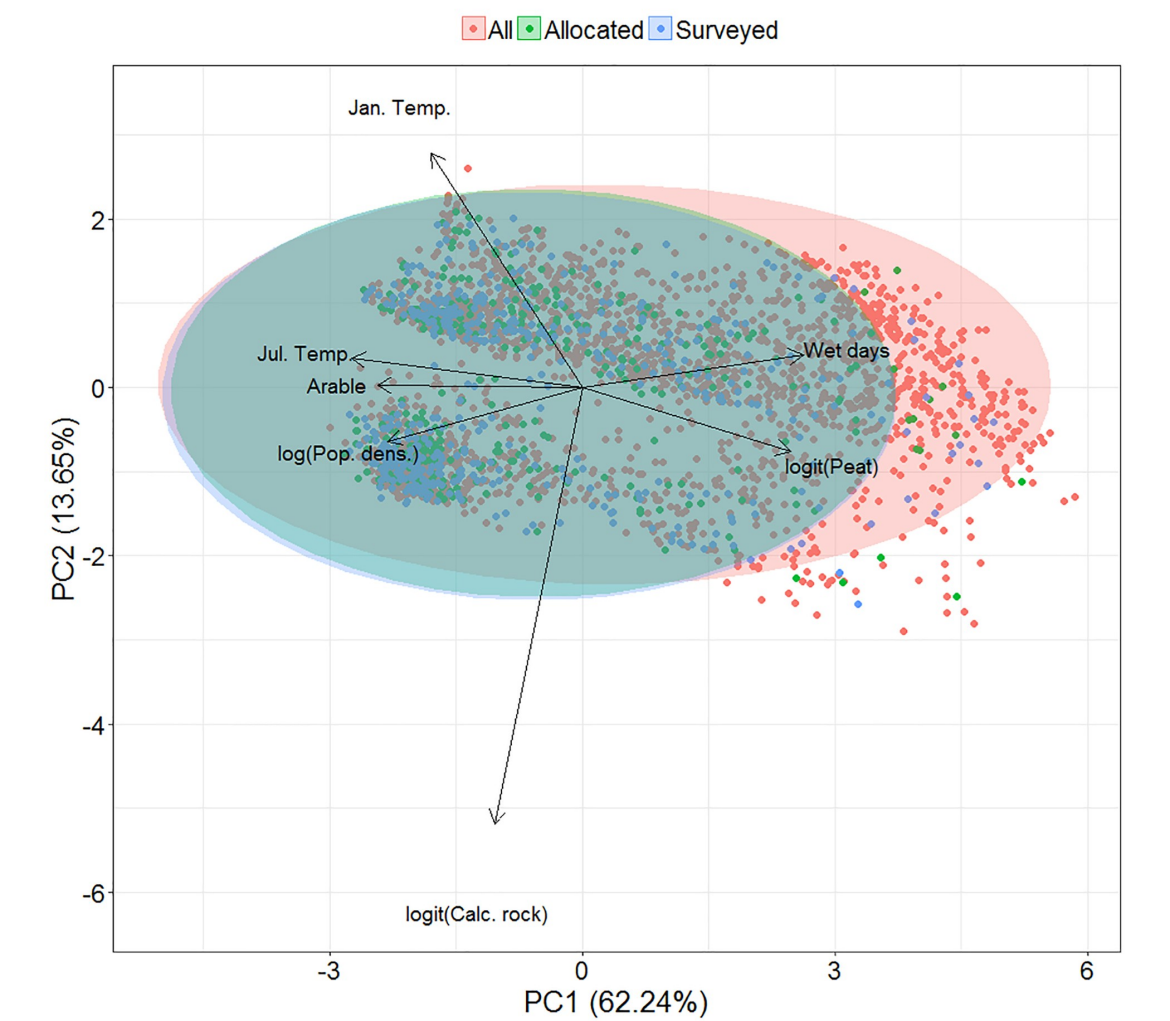
The environmental space covered by NPMS monads, divided by survey status. A principal components analysis of the UK environmental space covered by NPMS monads, subdivided by their status as either having been allocated to a surveyors or allocated and surveyed. Ellipses represent 95% confidence intervals. Sources for environmental data are given in the S1 Table.

Fig 6 illustrates the placement of all released, volunteer-allocated and volunteer-surveyed monads according to the first two principal components of the environmental space of our ordination. The PCA indicates that, although there is little difference in the environmental coverage of allocated and surveyed monads, these are clearly unrepresentative of the complete set of released monads, with monads in the more humid, peat-covered parts of Britain and Northern Ireland being under-represented in terms of volunteer interest and activity. This reflects the low relative representation of some upland and acidic habitats indicated by Fig 5.

### Scheme power

**Fig 7:**
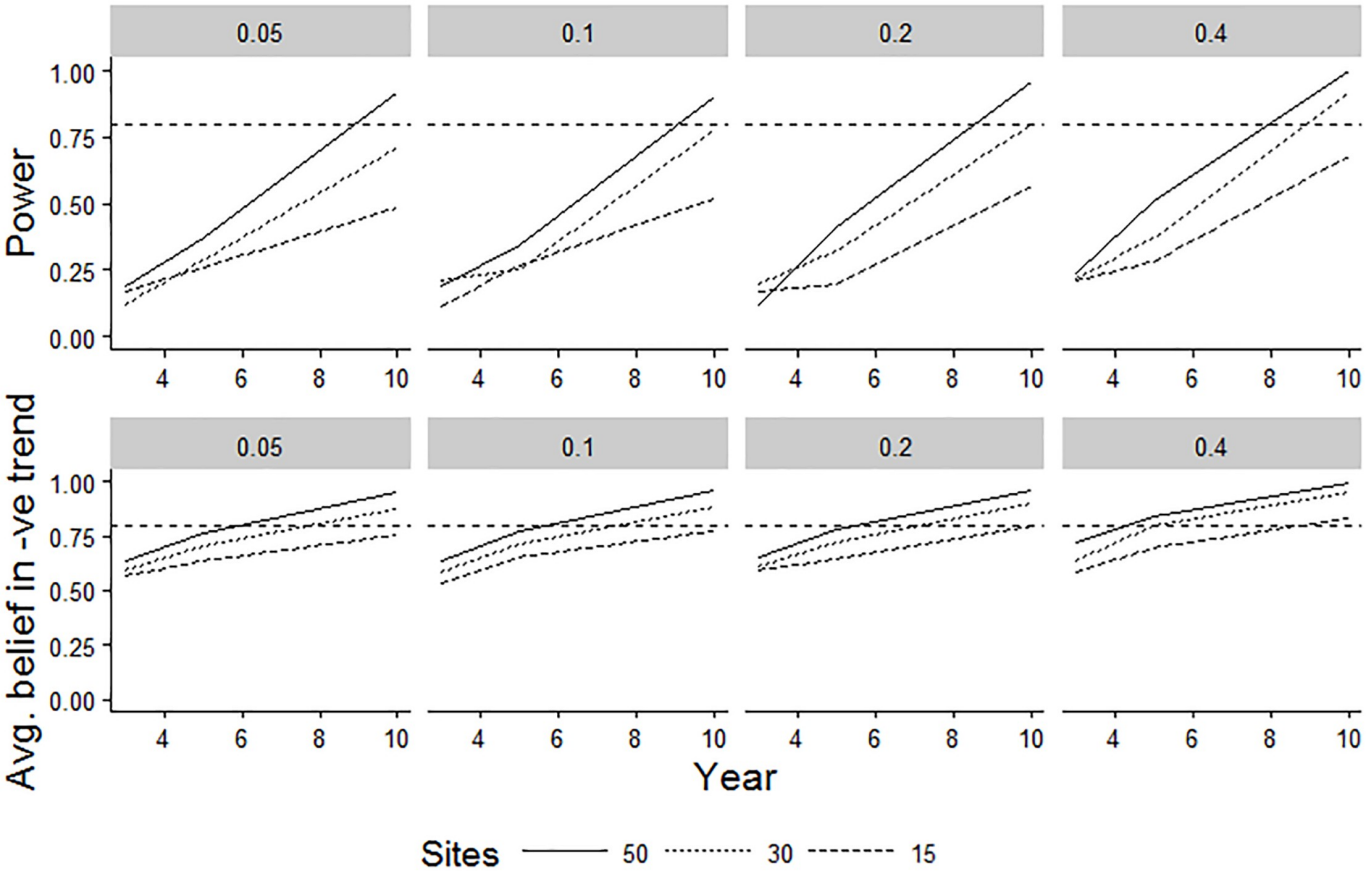
Comparisons of two alternative models of power for a single species’ decline. The underlying simulated decline was 30% per year over 10 years. Different columns represent different starting proportional covers of a species within a plot. The top row represents the results from a classical frequentist power analysis, using maximum likelihood parameter estimates of a simple linear model fitted to interval-censored data (Model 3 of [65]). The bottom row represents the average belief in a declining trend of any size resulting from a Bayesian hierarchical model fitted to interval-censored data. Interval-based data censoring was based on an approximation of the Domin scale in both cases. Trend line formatting relates to the number of modelled sites (n = 15, 30 or 50). The broken horizontal line represents the typically desired level of 80% power; although this is not appropriate for a Bayesian model, it is also included for this model-type to help assist with visual comparisons between the different scenarios.

Fig 7 illustrates representative frequentist and Bayesian model outputs for scenarios simulated with a 30% annual decline in a species’ abundance (recorded on an interval-censored scale). The most striking result is the dependence of conclusions on the mode of inference used (i.e. frequentist versus Bayesian). The Bayesian summary (Fig 7) represents the average belief in a negative trend given the (simulated) datasets; the frequentist power curve (Fig 7) gives the proportion of times the null test hypothesis of no decline is correctly rejected. Both models give standard power analysis results, in that a correctly diagnosed decline becomes more likely with time, and with an increase in the number of sites (Fig 7). Declines in cover are also more easily detected from a higher starting abundance [79]. Our results (Fig 7) [65] also show that a Bayesian model is likely to support belief in a decline at an earlier stage than a frequentist model, although this depends on how posterior distributions are summarised.

### Do NPMS species capture ecological gradients?

**Fig 8:**
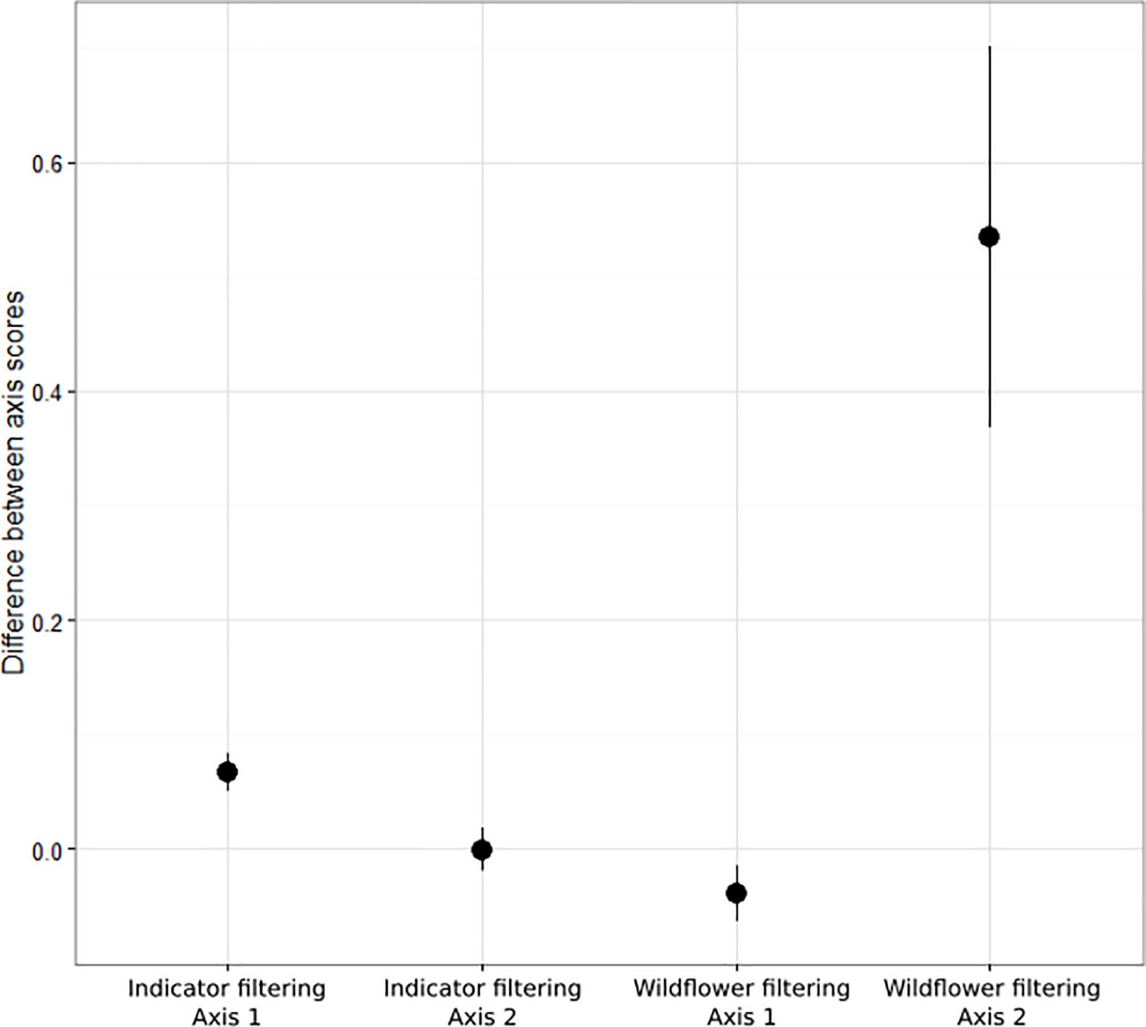
The loss of information on ecological gradients resulting from indicator species survey filters. Mean and 95% confidence intervals for the paired differences between DCA axis 1 and 2 scores for Countryside Survey plots before and after filtering by NPMS indicators or wildflower species.

The comparison of DCA axis scores from the full Countryside Survey 2007 dataset with their corresponding NPMS indicator species-filtered paired plot axis scores indicated that the major gradients represented in the full dataset [70] were retained. This suggests that the NPMS indicator species capture information on the main axes of ecological and environmental variation in the plant communities investigated (Fig 8). Further filtering of the Countryside Survey plots to retain only NPMS wildflower species, however, indicated that ecological signal related to DCA axis 2, representing succession and disturbance, was not so well retained (Fig 8). Along this axis, the NPMS wildflower filtering moved the mean axis score for the plots by 6.6% of the total length of the axis. This represented a shift towards taller vegetation, and it is likely that this reflects the reduced representation of grass species at this level that in general are characteristic of grazed, mid-successional vegetation. DCA axis 1 (fertility), however, was still well captured by NPMS wildflower species.

### Assessing potential recording biases

**Fig 9:**
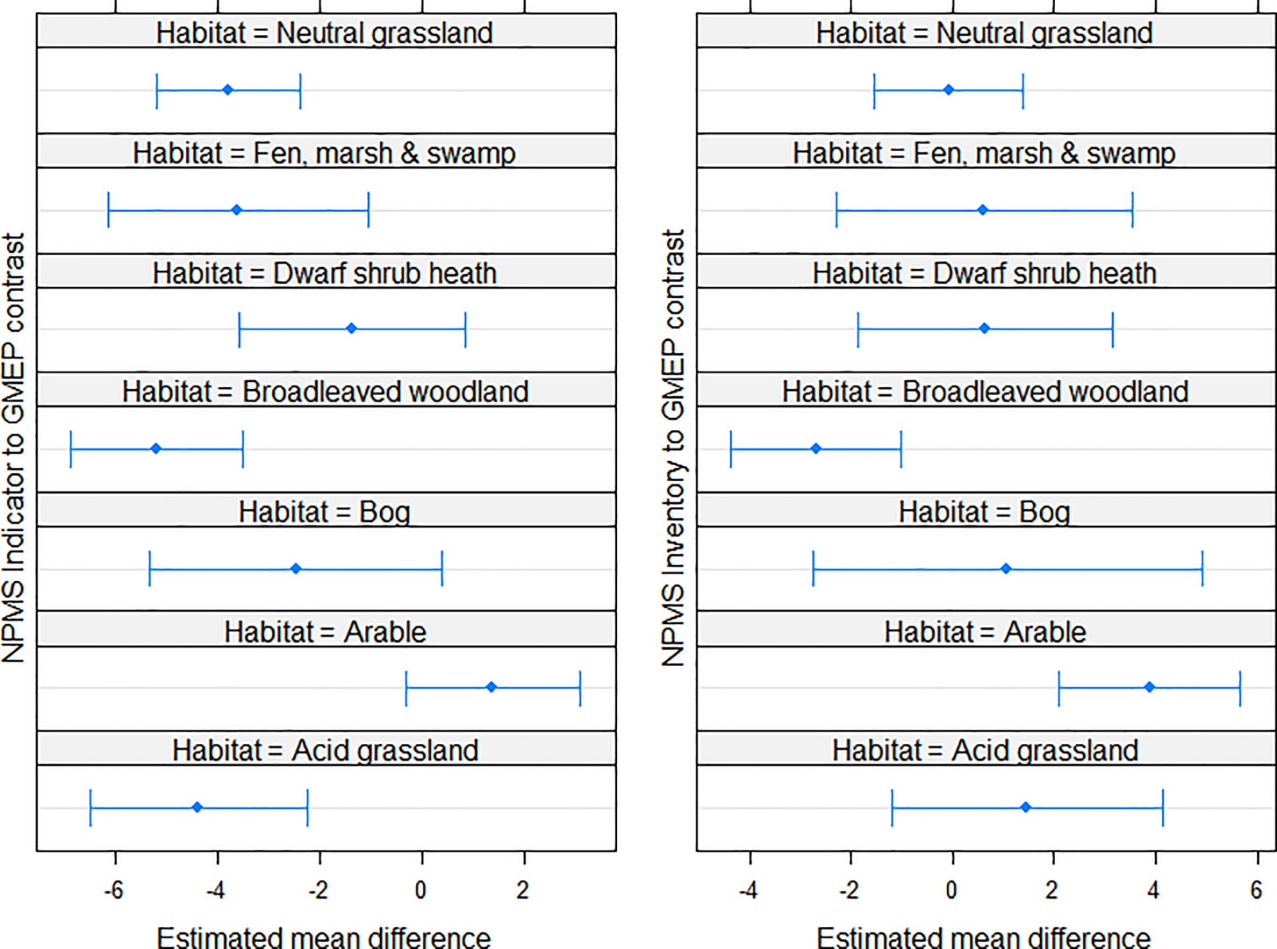
Comparisons of NPMS indicator and inventory richness in Wales compared to GMEP survey data. Broad habitat-based contrasts for NPMS indicator and inventory plots compared to their GMEP comparators (estimated mean difference with 95% confidence intervals). Note that all comparisons are based solely on presence of NPMS indicator species, irrespective of plot type. Such a constraint ensures that records made in all plots are drawn from the same list of plant species; all differences should therefore be due to effort or bias, rather that systematic differences in the species sought by recorders.

No overdispersion was detected in our Poisson GLMMs: GMEP vs NPMS indicator plots (chi-squared = 537.6; residual d.f. = 608; *P* = 0.98); GMEP vs NPMS inventory plots (chi-squared = 634.6; residual d.f. = 613; *P* = 0.26). In neither case did the model residuals show any indication of remaining spatial autocorrelation (Moran’s *I P* values = 1 and 0.99 respectively). The results of the global models are given in S5 File. Across the majority of habitats analysed, NPMS indicator plots were typically less species-rich then GMEP plots, whilst NPMS inventory plots tended to be slightly more species-rich on average (Fig 9); however, interactions between plot type (GMEP/NPMS) and habitat were significant in both cases (S5 File).

## Discussion

This paper has outlined the process through which a new UK plant monitoring scheme was designed, implemented, and assessed for its ability to achieve a variety of goals. As well as being created to detect and provide for the interpretation of changes in semi-natural habitat quality through the use of plant indicators, a significant part of scheme design was focused on providing a relatively simple and enjoyable survey method that would be attractive to a wide range of volunteer plant recorders, and which would also serve to develop expertise [40,41]. The inclusion of volunteers in scheme design, and frequent consultations, was a key part of the process, and resulted in numerous initial researcher-led proposals being adjusted or amended. The result is a monitoring scheme that is flexible for volunteers, but built to a rigorous specification.

### Monad uptake and environmental gradients

Our ambitious uptake target of 2000 monads was based partly on the numbers achieved in other successful UK structured monitoring schemes [23,24,80], but also to ensure that released monads were moderately abundant throughout the landscape, and available to volunteers without excessive travel (this was often cited as a constraint to participation by volunteers during scheme development). Stratification by region also serves to promote relatively even geographic coverage, however, this strategy also meant that areas away from human population centres also received monads, weighted, as in other regions, towards larger areas of nationally rarer land cover types. Reconciling the desire to produce unbiased assessments of priority habitat types with that of minimising volunteer travel is a frequently encountered problem in citizen science-based structured monitoring (e.g. [81]). Our analysis showed that, despite no difference in the environmental characteristics of monads requested by volunteers and those ultimately surveyed by them, those environments left largely unrepresented by volunteer activity were more likely to be wetter places with larger areas of peat, i.e. the UK uplands in the north and west. Analyses of the influence of regional population density on monad allocation and survey supported this conclusion, with the probability of both allocation and survey clearly increasing with population density. This conclusion held even after accounting for the clear spatial clustering of the population of the UK [82], suggesting that the influence of population density on survey participation is real, and not merely a correlate of another spatially structured covariate.

### Scheme power

The clearest practical conclusion from our analyses of potential scheme power was that, regardless of the average starting abundance across plots, Bayesian analyses are likely to result in a strong belief in a real decline for a single species after at least 8 years and 30 sites worth of data for a given species. The frequentist statistician might be left waiting a little longer before being persuaded that a null model of no decline was unlikely. For our Bayesian analyses, we averaged across a belief in a decline of any size, although other thresholds could be used. For example, Brooks et al. [83] created separate summaries for declines of different magnitudes in their assessment of lapwing (*Vanellus vanellus*) population declines. However, the conclusions from both modelling exercises are dependent on our input assumptions, such as the variance hyper-parameters used to simulate the species’ data modelled (see also [84]). We note that when species’ trends are aggregated to create indicators [85], then the power to detect an aggregate trend will increase according to the degree that the underlying trends agree i.e. common trends across species will increase power, because this is logically equivalent to an increase in the number of sites with similar trends monitored. Conflicting trends across species will tend to decrease power, because this is equivalent to increasing variance across sites.

### Habitat representation

Comparisons of NPMS habitat types catalogued by land cover GIS datasets in released monads with those habitats reported from the field were not suggestive of any major discrepancies, although patterns that are likely linked to monad uptake patterns were apparent. In both the mapping data and the survey returns, broadleaved woodland, hedges and scrub and dry grassland were the most frequently recorded habitats, although mapping data for the released monads suggested that current volunteer effort is under-representing broadleaved woodland habitats. This may be due to the lower survey levels in the north-west of the UK and/or because of the frequency with which private woodlands are encountered in the lowlands. Coastal habitats appeared to be slightly over-represented in surveyor returns, which, conversely, may be due to their typically open access status. Other apparently under-represented habitats included marsh and fen, native pinewood and juniper scrub, and rock outcrops, cliffs and screes. The first and third of these are habitat types that may often be hard to access in the landscape, whilst the lack of samples of the second is almost certainly due, again, to lower survey effort in the uplands coupled with more challenging access.

### Indicator species effectiveness

Our selected indicator species retained ecological signal related to the two main environmental gradients identified in an analysis of a large set of quadrats collected for a periodic, professional surveyor-based, unbiased, wider countryside monitoring scheme [57]. Our indicator species were subject to additional filters on ease of identification, within-habitat patch abundance, and landscape-scale frequency. The retention of gradients representing substrate fertility and succession after this filtering suggests that, across common UK habitat types, broad ecological patterns are well captured by common, locally abundant species. This is not unexpected in a country with a relatively species-poor vascular plant flora, but a geologically and climatically diverse landmass. Further filtering down to the smaller set of ‘wildflower’ level species did begin to degrade ecological signal, with the DCA axis representing succession losing part of its information content. Promoting surveyor progression from wildflower to indicator level is a stated aim of the scheme [41], and this assessment provides ecological support for the desirability of this goal.

### Recording biases

The comparison of a subset of NPMS data to a second independent survey (GMEP) conducted by professional surveyors is a good test of the potential recording bias within the NPMS. The results obtained suggest that, on average, plots recorded at the indicator level had lower recorded species richness than their GMEP counterparts, but that plots recorded at the inventory level appeared more species-rich on average, although this varied by habitat. The explanation for this finding is likely to be that NPMS indicator-level recording will be done by those volunteers less able to confidently census all species: these surveyors are, on average, likely to have recorded fewer indicator species than were actually present. This conclusion is supported by the fact the grassland habitats showed some of the clearest differences from the GMEP plots: the presence of grass species in the NPMS indicator list for these habitats, as well as habitat management (grazing or mowing), are both likely to have led to a lower rate of detection by volunteers with less experience of recording more challenging plant groups cf. [86]. The cause of the opposite, but less striking, bias in the NPMS inventory plots is not so clear, but may be due to surveyor decisions around within-monad plot placement (recall that surveyors may fall back on subjective plot placement if the primary, unbiased, methods fail due to land accessibility or other reasons). No obvious bias relating to broader landscape context was detected.

### The implications of bias

Pescott et al. [8] outlined the types of bias that were considered during the design of the NPMS. The current paper has investigated the subsequent reality of volunteer-based, small scale, plant monitoring using the first two years of data collected by our surveyors. As for many other such schemes [20,23,81], low coverage in the uplands of the UK will present challenges when attempting to derive trends for habitats and species confined to these areas: it may take some time to achieve the numbers of plots indicated by our power analysis; indeed, for some species restricted to very rare, or inaccessible, habitats, it may not be possible to report robust trends at all. The NPMS, however, has constructed strategies for dealing with these issues, e.g. by seeking to embed the scheme within the monitoring and volunteer outreach programmes of nature reserves and national parks, although professional ‘top-up’ surveying may also be considered in the future (cf. [87]; S6 File).

Biases at the smaller scale, e.g. positive and negative errors of plant identification or subjective plot placement, may also affect the ecological interpretation of monitoring data [88–90]. The results from the current investigations highlight areas where more detailed quality assurance will be desirable, as highlighted elsewhere [91]. For example, although comparisons using our land-cover based weightings indicated a similar set of monads were sampled by the NPMS and GMEP, the, on average, richer plots recorded at the NPMS inventory level may indicate biases resulting from subjective plot placement and/or issues relating to land access. Despite similar land covers to the GMEP monads, it may be that surveyed NPMS monads contain greater areas of protected land such as open access nature reserves. As for the findings reported here, future quality assurance exercises will also provide useful lessons for the scheme in terms of both volunteer support and future data analysis.

### Maintaining volunteer interest and enthusiasm

Any citizen science-based structured monitoring scheme with long-term aspirations has the clear and obvious goal of maintaining interest and a sense of personal investment by volunteers. Although volunteer-based ‘opportunistic’ plant recording in Britain and Ireland has an exceptionally long history [8], structured schemes with more prescriptive methodologies, in terms of where volunteers go and what they record, are likely to have to work harder to maintain engagement and participation [92]. Demanding precise and repetitive action from volunteers in restricted areas, as opposed to the more exploratory and free aspects of opportunistic or Atlas recording [67], may create greater challenges for scheme recruitment and retention e.g. [93]. However, the close link between surveyor activity and outputs typically characterised by structured monitoring e.g. [80] may offer one advantage over opportunistic recording, in that data flows, visibility, usage and impact, in terms of environmental outcomes, may all be clearer to the volunteer e.g. [80,87,94]. The ethnographic research of Ellis & Waterton [95] highlighted this possibility, documenting an example of a field recorder engaged in a relatively unstructured biological recording scheme feeling alienated from the subsequent uses of their data; we do not know how widespread this feeling might be.

Ensuring that volunteers are well supported plays a vital role in maximising participation in citizen science [92,94]. The feedback from volunteers detailed above resulted in numerous improvements to the draft guidance material issued during our transitional year (2014), resulting in clearer instructions, and diagrams to illustrate key field techniques [58]. Improvements to materials also led to the production of an identification guide for all indicator species, and better links between the identification guide and the species lists for habitats provided to volunteers (www.npms.org.uk/content/resources). In addition, a programme of training workshops is provided each year covering the NPMS methodology, online data entry, and species and habitat identification, with the balance between these provisions changing each year based on an end-of-year email consultation with participants.

A peer mentoring scheme has also been developed and implemented. This involved the production of a support ‘toolkit’ for mentors and the delivery of further training. The role of the mentors is to boost confidence, support local networks of volunteers, and improve accuracy and consistency of survey technique. However, we also hope that this approach goes some way towards creating local networks of surveyors, enabling local interpretations and uses of data [95,96]; indeed, such a perspective has been emphasised in scheme communications alongside national monitoring objectives [41]. The first cohort of 19 mentors were active during the 2016 field season and were able to help over forty volunteers with their enquiries. It is hoped that this number will increase as peer mentoring becomes further embedded within the scheme, mirroring the support networks long used by other natural history societies and recording schemes [2,97].

National implementations of structured biodiversity monitoring by volunteers will naturally be shaped by local traditions and infrastructures supporting biological recording, and it is difficult to provide direct recommendations for other countries in ignorance of these. We hope, however, that both the general and specific paths taken by us during the development of the NPMS will still provide a set of ideas for other countries to experiment with and consider.

## Conclusions

It has been observed that it may not be possible to “unilaterally define” success for citizen science programs [98]; from our perspective as scheme organisers and analysts, success is clearly the continuation of the scheme with adequate volunteer participation to enable the creation and use of reliable and scientifically robust indicator metrics for our habitats and species of interest. This success, however, will necessarily entail many other successes in encouraging, supporting, and improving the skills of botanists engaged in the scheme, as well as in highlighting local conservation and ecological stories, and ensuring that the scheme provides adequate opportunities for volunteer development of many different types. Lawrence [96] found that her recorders underwent personal transformations in spite of participation in largely top-down projects. Establishing an ongoing dialogue with participants is a key part of our ensuring that volunteers have the opportunity to point out where our focus on their personal development could be improved, for example through our peer mentoring scheme, our species and habitat identification workshops, through regular opportunities for feedback and more formal reviews, and through newsletters and articles in more popular journals e.g. [41]. Finally, and in addition to our recognition of the potentially diverse needs of our participants, a number of measures are being put in place to ensure that the NPMS is embedded within different government conservation and land management organisations. For example, embedding the survey in large land-owning organisations through staff training and development will reduce scheme vulnerability, and should also help to improve coverage of the remoter parts of the UK. The position of the NPMS within a much broader network of plant recorders and conservationists in the UK will also continue to provide a diversity of perspectives and skills for the scheme to build upon [2,8,67,99]. We also expect, and hope, that all of our stakeholders will continue to challenge us to provide the best evidence base possible for plant conservation in the UK.

## Supporting information

S1 Table UK environmental data used in the PCA of NPMS monad environmental space. (DOCX)

S2 Table NPMS species. A complete list of NPMS species and species aggregates with their associated fine-scale habitat, status (positive/negative), and level (indicator/wildflower).
(XLSX)

S1 File NPMS habitats tables. Two lookup tables are provided: NPMS broad/fine-scale habitat to the British National Vegetation Classification; and, NPMS broad/fine-scale habitat to EUNIS.
(XLSX)

S2 File Volunteer field trial summary report. (PDF)

S3 File Web consultation questionnaire questions. (XLSX)

S4 File Selected volunteer feedback from the field trials and online consultation. (DOCX)

S5 File Assessing potential recording biases global model results. (DOCX)

S6 File Promoting the NPMS. A brief overview of the approaches taken to promote the scheme, pre- and post-launch (2014–2016).
(DOCX)
