# Supplementary material for: The design, launch and assessment of a new volunteer-based plant monitoring scheme for the United Kingdom
Source: PLoS One. 2019 Apr 26;14(4):e0215891. doi: 10.1371/journal.pone.0215891 (PMC6485706; doi:10.1371/journal.pone.0215891)
Supplement: S1 Table — (DOCX) [file pone.0215891.s001.docx]

**S1 Table**

UK environmental data used in the PCA of NPMS monad environmental space.

| Environmental factor(s) | Source |
| --- | --- |
| January mean temperature and July mean temperature (both 1981-2010) | Met Éireann (Ireland); Met Office (GB) |
| Mean number of wet days per year (1981-2010) | Met Office (UK) |
| Arable land | ‘Arable and horticulture’ cover class, Land Cover Map 2007 (UK) (Morton *et al.* 2011) |
| Peaty soils | ‘Bog’ cover class, Land Cover Map 2007 (UK) (Morton *et al.* 2011); Derived Irish Peat Map Version 2 (Republic of Ireland) (Connolly & Holden 2009) |
| Calcareous rocks | BGS Parent Material Model Version 6 (Great Britain); BGS 1:625,000 Bedrock Geology (Northern Ireland). The original map (Blockeel *et al.* 2014) is based on bedrocks with CaCO3 contents classified as High (e.g. chalk), Variable (high) (e.g. interbedded limestone and mudstone beds) and Moderate (e.g. dolomitic limestone, calcareous mudstone) |
| Population density | Gridded Population of the World, Version 3 (CIESIN 2005) |
