# Supplementary material for: The design, launch and assessment of a new volunteer-based plant monitoring scheme for the United Kingdom
Source: PLoS One. 2019 Apr 26;14(4):e0215891. doi: 10.1371/journal.pone.0215891 (PMC6485706; doi:10.1371/journal.pone.0215891)
Supplement: S2 File — (PDF) [file pone.0215891.s004.pdf]

NATIONAL PLANT MONITORING SCHEME FIELD TESTING REPORT – SEPTEMBER 2014

KEY RECOMMENDATIONS

Protocols around plot selection

- Volunteers will be allocated plots to sample but it is essential that a clear protocol for self selecting plots is also provided. Accessibility is likely to be a significant issue, perhaps greater than has been factored for at this stage, and it is vital that a clear protocol empowers volunteers to make good decisions that inspire confidence in them and deliver the robustness of data the survey demands.
- Have a flexible approach to the balance of square and linear plots allowing volunteers to increase the number of linear plots they do should they be unable to find three accessible plots in target habitat. This will help counter some of the accessibility issues faced above.
- Encourage volunteers to survey as many habitats as possible. This will mean clear guidance for volunteers whose accessible plots may all fall within one habitat. This may also mean an increase in the proportion of volunteer selected plots.

Plot size

- Plot sizes should be varied according to habitat with 5 x 5m or 10 x 10m for square plots, and 1 x 25m for linear plots, provided this is suitable and appropriate across habitat types.

Levels

- The way in which levels are communicated needs consideration in particular with regard to how the species lists are talked about. Talking about 400 species in total may seem a lot to volunteers and the fact that volunteers are only encountering a subset of the 400 per habitat should be stressed.
- Stronger guidance as to what the inventory level encompasses is needed.

Information provision for volunteers

- The guidance for carrying out the survey and entering data online needs substantial improvements. There also need to be modifications in the online data entry process to facilitate volunteers using this.
- Use the best quality data available when providing volunteers with maps. Every effort needs to be taken to ensure that the language describing habitats on maps is the same as that used in the guidance.
- Ensure volunteers are aware of likely time commitments and are also aware that these will reduce in subsequent years. Advise volunteers that they undertake a separate visit for identifying plot locations prior to making subsequent visits to record plants.
- Ensure information about survey purpose and species selection is communicated well to avoid volunteer disappointment with regard to numbers of species encountered or modify lists to increase species encountered. In addition optimise the inclusion of species that volunteers may encounter without jeopardising the information being gathered.

Species

- The data gathered in this field testing phase suggests that for some habitats volunteers encountered insufficient species to record. This is something that needs addressing especially given the increase in effort required for participation in the new survey.

1.0 AIM OF FIELD TESTING PHASE

The aim of the field testing was to explore further and understand volunteer experiences of different plot selection methods and different plot sizes. The ability of the different plot selection methods to locate plots within accessible target habitat was explored as was the number of species recorded in different habitats. In addition the accuracy of maps with target habitat marked on them was explored; most participants being supplied with either Land cover maps or Priority habitat maps.

2.0 APPROACH

Participants from Plantlife's current pool of 3500 volunteers registered to participate in the existing Wildflowers Count survey were asked to come forward to field test 4 proposed plot selection methods and 2 plot sizes as well as two map types; Land cover map and Priority Habitat map. Ninety three people came forward. All field test participants had at least 1 year of experience in the survey with 80% having 3 or more years of surveying illustrating the experience of this self-selected pool. A separate consultation survey solicited responses from the pool of 3500 volunteers. Three hundred and sixty six responses were collected. A separate report is available however in the results occasional comparison is drawn between the field test participants experiences and those who responded to the consultation survey.

Each field test participant was assigned a survey method; map and plot size – thereby gaining a combination of factors to test as shown in the table below. 38 participants reported they had completed the survey using one of the methods that had been allocated to them. The participants carried out the survey in the square that they know and survey as part of the Wildflowers Count survey. Only **1** participant recorded in upland (land over 300m) and therefore there is a bias associated with this report that perhaps does not reflect all implications of the proposed new survey.

Table 1: Allocation of map, plot selection method and plot size

**Plot selection method**		**Map**		**Plot size**	
Gridded	6 (25)	LCM	15 (46)	5x5m	23 (47)
Random	12 (24)	PH	20 (43)	10x 10m	13 (46)
Targeted	5 (21)	Phase 1 (Wales only)	1 (4)		
Volunteer selected	13 (23)				

3.0 RESULTS

3.1 Which survey level did participants carry out?

The volunteers were able to select which survey level to do. Nine (25%) selected indicator level plots, whilst 44% recorded at the wildflower level and 31% at the inventory level. It is encouraging to note the number recording at inventory level suggesting high levels of expertise. However a number of people who stated that they completed the inventory level also stated they could not record, for example, the grasses because they had been grazed. The indicator level plots were new this year so poorer take up could be reflected by this. It is extremely encouraging to note that 52% of participants stated they would feel comfortable in moving to the next survey level up – although it is worth bearing in mind the fact that this self-selected pool may be more motivated than other existing volunteers. It is suspected that in many cases people state they are recording inventory

level (all species present) when in actual fact they are recording all species present they can identify – so the ramifications of this should be considered.

It is clear that there needs to be some thought around the levels and it is worth considering whether modification of species at the indicator level would increase participation at this level. For example are there alternate easier to identify species that still meet the list requirements in terms of indicating quality of habitat.

Another factor that could be considered is the removal of the wildflower plot level – or phasing out so as to act as a push factor for migration to the indicator level. The levels and number of species within each level are communicated is important – as no participant is likely to encounter all 400+ species and talking about the number of species is possibly a barrier. It is preferable to talk about average number of species per habitat.

3.2 How accurate were the maps that were supplied to participants?

The intention of the NPMS is to ensure that surveyors are carrying out their surveys in semi-natural habitats and one of the ways of doing this is to supply them with maps with target habitats indicated. Two participants stated that their map information was completely wrong and therefore did not correlate with the map e.g. NZ1600. Nearly 40% of participants stated that there was poor correspondence between map and ground. Only 3 people (8%) felt that all the habitats on the ground correlated with those shown on the map.

The responses indicate that existing data on habitat type clearly is not as accurate as may be envisaged and that the new survey therefore has a good contribution to make in terms of both picking up new habitat but also identifying where what is thought to be existing target habitat has changed. However this is an additional burden on surveyors and given the likelihood of a merger of habitat types at the surveyor end may not be something that is within the scope of the survey.

Further examination comparing responses from those who were provided with LCM (Land Cover Maps) maps as opposed to those provided with PH (Priority Habitat) maps, Figure 1 illustrates very clearly that the LCM maps were where volunteers reported the least correlation between map and ground. This evidence supports the idea of providing volunteers with the best quality maps available although it must be acknowledged that this will mean volunteers in different countries having different maps.

Participants' comments with regard to mapped habitat and ground correlation were also collected and a selection shown in Figure 2, (map type provided in brackets) – illustrating the kinds of situations encountered. It is worth bearing in mind that this pool of field testers are likely to be more experienced and knowledgeable than the general pool of surveyors and that therefore illustrating the shading on maps in aggregated habitat types may be preferable in the future. There is some suggestion that the guidance may need to be clearer with regards to surveyors understanding that irrespective of the habitat type the map may attribute to a plot if that plot is a semi-natural habitat and falls into one of the habitat descriptions in the NPMS then it should be surveyed.

Figure 1: Volunteers perceptions of map and ground correlation with regard habitat

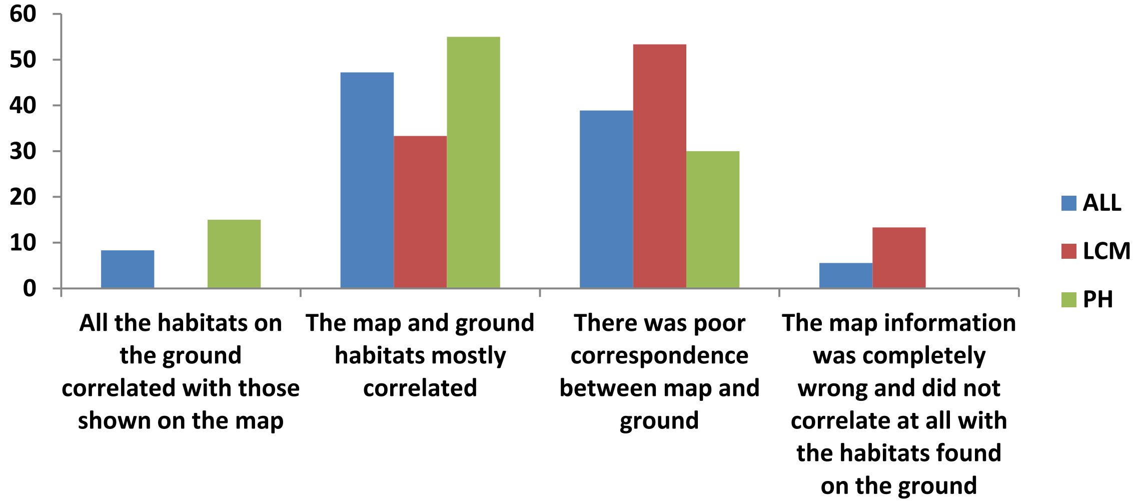

Figure 2: Volunteers experiences of map and ground correlation

Heather and acid grassland were indistinguishable which were little different from rough grassland. One area of improved grassland I did not find. (LCM)
Significant under recording of lowland meadow. (PH)
Some of the deciduous woodland marked on the map was pasture/meadow on the ground. The coniferous woodland was less extensive than shown on the map. Also the river/stream was a reservoir on the ground. (PH)
Acid grassland (plot 1) was actually a deciduous sloping woodland which is heavily disturbed by walkers with paths running down to a caravan site. Plot 3 Heather grassland was rough grassland with no access containing bracken, gorse and nettles along margins. (LCM)
The landcover map was poor classing most of the habitat as rough pasture but in fact there is a mosaic of improved, rough and wet / bog habitat. (LCM)
One area described as deciduous woodland was stands of trees around dwellings in a residential park and the other area described as deciduous woodland consisted of rows of deciduous trees along water courses. Possible priority habitat was rows of trees planted in the residential park. The area of Biss Meadow Country Park was not mapped and consists of priority habitats, such as grassland, lakes, watercourses and hedgerows. (PH)
Traditional orchard - landuse changes have occurred since the map was produced (PH)
Neutral grassland now mostly arable, broadleaved woodland fairly inaccessible (LCM)
Some areas of broadleaf woodland that were scrub (LCM)
Indicated as neutral grassland but now mostly cultivated; neutral grassland nearby though (LCM)
Large areas of deciduous woodland no longer present (PH)
Area noted as deciduous woodland is now a limestone quarry with no access. (PH)
Downland (calcareous grassland) was shown as arable. Woodland was not accurately shown. (LCM)
No heather grassland. All the centre of the 1 km square is deciduous woodland and not calcareous grassland. (LCM)
Rough grassland was not present in the areas marked on the map - the shaded areas for the habitat on the map were mostly paved, hard standing, entrances or the road. Habitat 1 was a pine plantation not deciduous woodland. (LCM)
The broadleaved woodland was a cemetery- well mown. (PH)
1 should have been bracken and was a private planted wood; 2 was correctly dry heath; 3 was broad-leaved woodland not marshy grassland; 6 was acid grassland not scrub; 7 was acid grassland not wet heath

3.3 What was the impact of allocated plots on time taken in the survey square?

Participants were asked how much time they spent in their square – for one visit only. Figure 3 shows the time taken for field test participants, as opposed to those carrying out the survey and self-selecting plots or pathways (consultation) and those self selecting plots (consultation – without pathways).

Clearly the new methodology requires people to spend more time in their square than the existing Wildflowers Count survey. This can be explained by two factors: 1) the difference between surveying plots as opposed to a pathway and 2) the time impact for locating allocated plots as opposed to self-selecting plots. It is worth bearing in mind when communicating with existing and potential volunteers about recruitment onto the scheme the time commitment necessary and explaining that once plots have been located in the first year the time commitment will reduce. This will help reduce resource wastage resulting from volunteer attrition. At the Juniper Hall residential the possibility of encouraging potential surveyors to do a reconnaissance visit to locate their plots was discussed and it is felt that advocating such an approach will minimise perceived surveying times.

Figure 3: Time taken in survey square

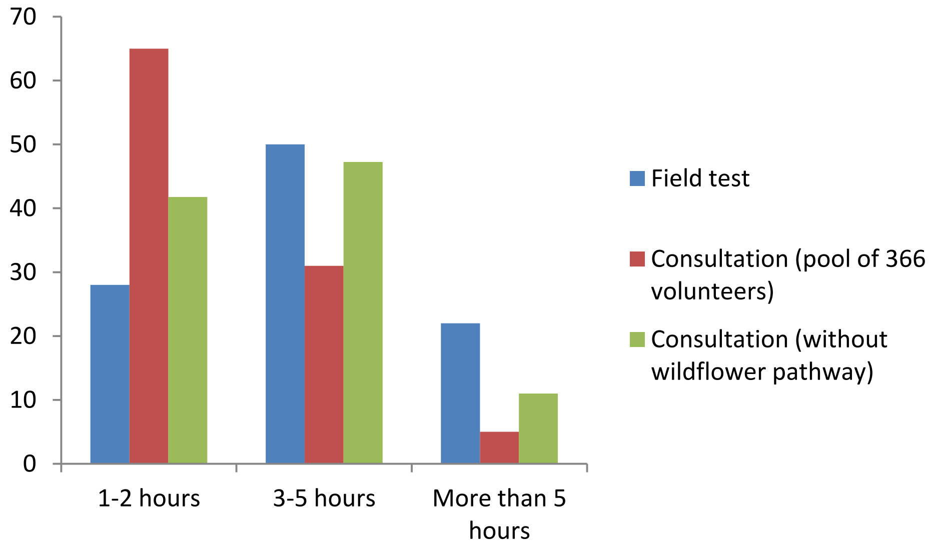

3.4 What was the impact of survey level undertaken on time taken in square?

As expected those recording at higher levels spent more time in their survey square. Clearly the more species being recorded the greater the length of time taken. The new NPMS requires many people to be participating at the indicator level. Figure 4 perhaps gives some idea of the increase in time that recording at this level may take as opposed to recording at wildflower plot level. Perhaps this is due not only to the greater number of species but also to surveyor confidence in identifying those species. It is suggested that any revisions to the species lists should bear this in mind as suggested above. In addition scope for participants to record at different levels 'a mix and match approach' could be considered.

3.5 What was the impact of plot size on time taken to complete the survey?

Participants were also asked how long it took them to survey a plot once that plot had been set up. All respondents stated to survey 1 plot took less than an hour with 60% stating that it took under 30 minutes. This is in line with experiences at the Juniper Hall residential. Surprisingly there was no recorded increase in time taken to record a single plot for those who were recording in 10x10m plots, as illustrated below in Figure 5. This probably reflects plot set up time and is borne out when total survey time is analysed by plot size, Figure 6. This is encouraging given the emerging evidence that suggests having 2 different plot sizes determined by the habitat being recorded is a preferable approach.

The data from the consultation survey (plots 5x5m) suggests people spent longer in plots than those participants doing the field test - although this can probably be explained by the fact that the measure for this cohort included time taken to set the plot up. It may also be explained by surveyor experience, participants in the field testing perhaps having more experience than the general pool of surveyors.

Figure 4: Length of time spent in survey square by level

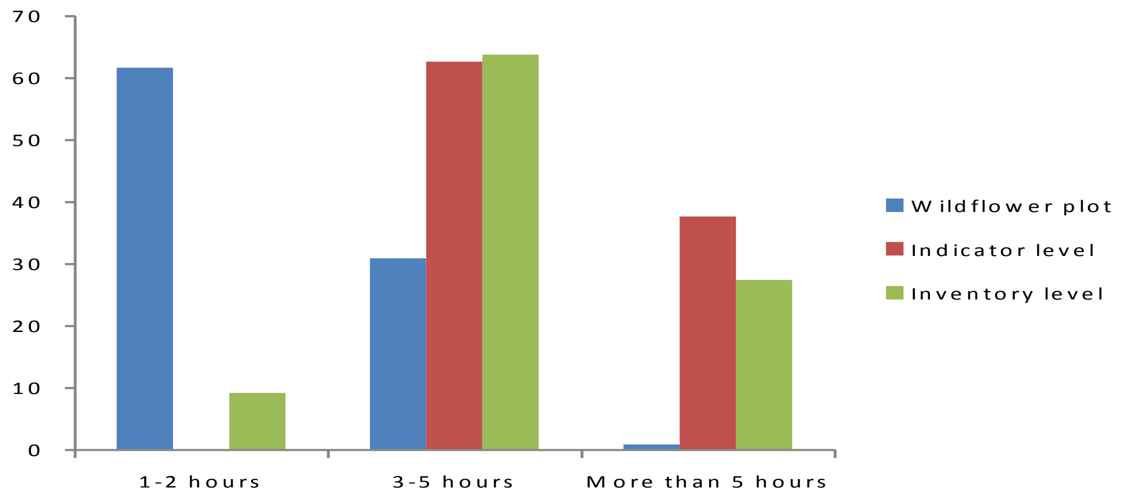

Figure 5: Time taken to record in different plot sizes (percent respondents)

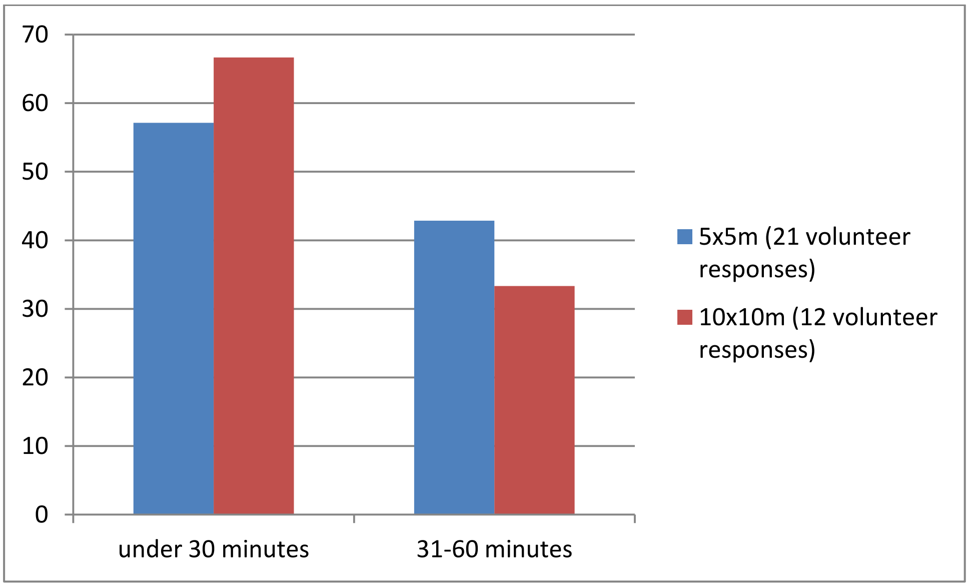

Figure 6: Time spent in square for different plot sizes

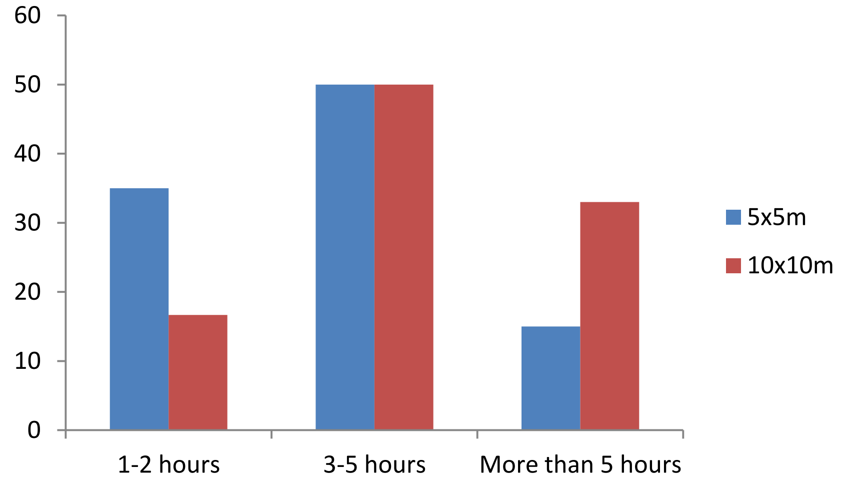

3.6 How many habitats were volunteers able to survey and did plot selection method impact on the number of habitats visited or the number of plots surveyed in mapped habitat?

One of the reasons for developing the methodology was to ensure that volunteers visited different habitats. One reason for this is to sustain volunteer interest as previous experience has shown that volunteers like to be able to record in different habitats.

Seventy percent of volunteers visited and surveyed less than two different habitats (excluding linear plots and ponds and flushes) using the mapped habitat alone. If volunteers were unable to access enough plots in mapped habitat they could self select plots in other target habitats that may not have been mapped. The ability to find and survey target habitats that do not fall into the shaded mapped areas does have some marginal, impact on increasing the number of different target habitats volunteers are able to survey. These results are probably reflective of the fact that the majority of the respondents were surveying in lowland in more populated areas and therefore encountering accessibility issues for some habitat parcels. There is perhaps a need for a clear decision as to whether the new NPMS will focus individuals recording multiple plots in the same accessible habitat thereby limiting people’s experience or will have strong, clear guidance that allows people to locate other target habitat that is unmapped within their square. The inaccuracies of mapped habitat have been shown above.

There is insufficient data to draw any conclusions about whether plot selection method limited or enhanced the number of plots visited in mapped habitat. However for gridded and targeted methods the average number of plots visited in mapped target habitat was 2.6, for random it was 1.3 (but this is a very small data set) and for volunteer selected 1.75. The data is therefore suggesting that a combination of plot selection method and mapped habitat did not adversely impact visits to target habitat compared to volunteer selected.

Figure 7: Target habitat surveyed in mapped and unmapped habitat

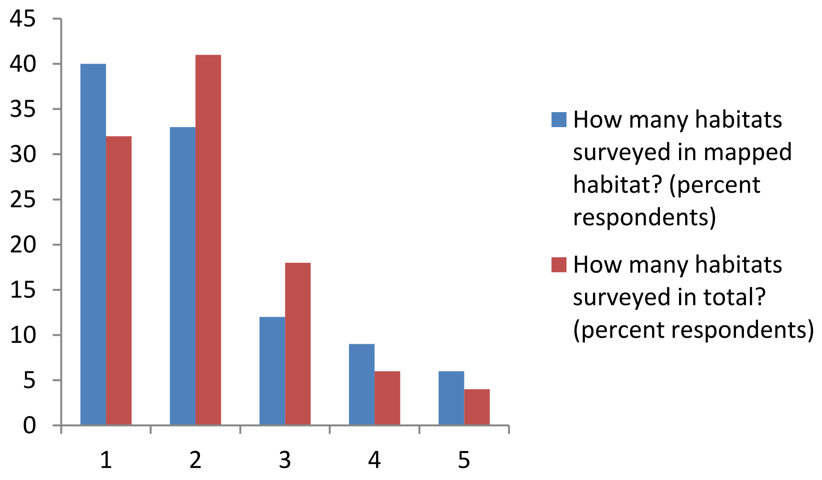

Participants who were using Plot Selection methods 1-3 were asked how many plots they had to visit before they had found and located three accessible plots in mapped habitats. Ten participants reported that they were unable to access 3 allocated plots in mapped habitats and this is confirmed by the fact that 34% of those doing plot selection methods 1-3 stated they had had to self-select plots. This shows the importance of clear instructions with regards to situating self-selected plots should there be a need.

3.7 Was the survey methodology sufficient in enabling volunteers to survey linear plots?

Using the methodology provided volunteers were asked how many linear plots they were able to find and survey. Just over 80% percent of participants surveyed at least 1 linear plot, with 43% surveying two. This is encouraging as it suggests that even if access to different target habitats for square plots is limited – reducing surveyor experience, most surveyors are likely to encounter additional habitats through locating linear habitat. Perhaps there is a need to consider greater flexibility as to the composition of square and linear plots to allow for those who may just encounter 1 accessible mapped habitat in their area.

Table 2 shows the number of surveys carried out in different linear habitats by the participants. The fact that all bar one participants were surveying in lowland areas explains the fact that screes have not been surveyed and rock outcrops were only encountered once and underlines the importance of ensuring that the new NPMS ensures squares in remote areas are surveyed thus providing information on these habitats.

Table 2: Linear habitat type and number of times surveyed

Linear habitat	Number of participants surveying
Hedgerow	10
Arable field margin	8
River	8
Road verge	6
Lake Margin	4
Rock outcrop	1
Cliff top grassland	0
Scree	0

It was suspected that hedgerows and arable fields would be the linear habitats most frequently surveyed. It is encouraging to note that a number of surveys were carried out along river margins as it had been a concern that this habitat could be underrepresented due to the fact that many river courses run through private land or the river bank is only accessible to angling clubs. It will be interesting to examine the first year from the NPMS and see which linear habitats are being surveyed and if necessary work to ensure that others are accessed and surveyed – perhaps through raising the profile of the survey amongst angling clubs for example, or through working to ensure adequate numbers of surveyors are recruited in remote areas who are confident in surveying in upland locations.

3.8 How long did it take to survey linear plots?

Fifty percent of participants stated that it took them under 30 minutes to survey their linear plot (60% for square plots) and 40% that it took them 31-60 minutes (which is the same as that reported for square plots). Three people stated it had taken them between 1 and 2 hours to survey a linear plot – no people reported this length of time for square plots. Influence of plot size could not be shown. At the Juniper Hall meeting possibility of extending length of linear plots in some habitats was discussed – clearly this will have ramifications on time spent by surveyors in square. Feedback from one participant involved in the field survey this year included the following statement.

> Having read the aims of the field survey, I was curious to know how 'plot size impacts on the species recorded' could be assessed if we were only being asked to do one particular plot size in our habitat. To this end we decided to complete our survey at both plot sizes, with the smaller plot included within the larger to see how much more information would be gleaned by enlarging the plot size (or lost by having a smaller plot size).
>
> We did this for the three woodland plots, the linear hedgerow and the linear field margin. From this survey, we felt that 5m x 5m was too small an area for a woodland plot and that more information could be gathered from the larger 10m x 10m plot. This may not be the case for habitats dominated by smaller plants, such as meadows. Maybe the nature of the habitat should dictate the size of the plot. For the linear habitats, extending the plot from 25m x 1m to 100m x 1m obviously included a greater variety of species, especially for the hedgerow but it became far more difficult to assess % cover as a 100m span is difficult to gauge.

3.9 Were the species lists sufficient to enable participants to record enough species for each habitat?

Surveys were carried out in nine habitats. Deciduous woodland and pasture/ meadow were by far the most frequently visited habitats, Figure 8. This is not unsurprising given the fact that this habitat is broad. It also perhaps reflects the accessible nature of many woodlands. There was surprisingly no different in average species recorded for wildflower plot and indicator plot levels. It should be noted that those recording at wildflower plot level recorded in 6 habitats whilst those at indicator and inventory level recorded in 8 habitats. The assertion that those recording at higher levels access a greater range of habitats was alluded to in the consultation report and is worth bearing in mind.

Note all calcareous species numbers indicated in bold recorded by single surveyor.

Figure 8; Which habitats were recorded? (square plots only)

	Deciduous woodland	Pasture / meadow	Wet grassland	Dry heath	Species rich lakes and ponds	Species rich fens / flushes	Calcareous grassland	Acid grassland	Wet woodland	Blanket Bog	Wet heath	Lowland rocks and screes	
Inventory level	12	5				2	3	1		2	2	1	
Indicator plot level	3	3	4	5	3	4	9						
	2	6	10				8						
	1	0											
	4	3											
	18	2											
		3											
		2											
		7											
Number of plots recorded	**5**	**8**	**2**	**1**	**1**	**1**	**2**	**0**					20
Average spp. per plot	2.5	1.5	7	5	3	4	8.5	0	0	0	0	0	**4.15789474**
Wildflower plot level	8	3			2	2		1	0				
	2	**6**						1					
	3	**8**						1					
	3	**6**											
	10	**9**											
	3	**8**											
	4	7											
Number of plots recorded	**7**	**7**			**1**	**1**		**3**	**1**				20
Average spp. per plot	4.7142857	6.7142857	0	0	2	2	0	1	0	0			4.35
Total plots	24	20	2	1	2	4	5	4	1	2	2	1	

3.10 To what extent could accessibility impact on the ability of participants to access the squares?

Participants could not always access plots that had been allocated to them. The participants who took part in the field testing knew their squares and therefore had a history of surveying in that square. Thirty two percent of participants stated they asked land owner permission, 45% stated they did not. Twenty four percent stated that the question was not applicable. This is partly explained by those self selecting plots.

Participants were invited to supply additional comments. The comments illustrate the importance of the local connection – participant and landowner; the interest amongst some landowners (farmers) and the interest from NGOS in the survey and data. This is something that should be acknowledged and harnessed in survey promotion – and has been addressed in the tender.

No problems unless shooting or stalking was planned. Landowners a little bemused.<br><br>attempted to seek permission for plot 2 but could not establish owner so plot relocated adjacent to footpath<br><br>HIWWT for Ancells Farm Nature Reserve - It is a public access area, but they appreciated my asking permission. Fleet Pond Ranger - gave permission for survey in restricted area as well as public area.<br>8<br><br>I had to get permission to access the area around the aerodrome, which was not problem as long as i had a Hi Vis vest. Also the area around the reservoir is accessed by a key I had to obtain from the Herts and Middx Wildlife trust with a £10 deposit. Again this was no problem, but a little more time consuming as the office is in St Albans.<br><br>marked plots 1 & 2 were on private land owned by someone I suspected would not allow access so didn't ask. Had also recently been mown too<br><br>Very happy to oblige, wanted survey data (National Trust)<br><br>I have been surveying these plots for six years with the full co-operation of the land owner. He is interested in what plant life is doing and I give him a report on the plants I find on his land each year. All my plots fall on his land.<br><br>I had to seek landowners permission last year for the plot I did, and was made very welcome, owner of the local Mushroom farm was interested and supportive<br><br>NT land. Previously shared Plantlife contact with warden<br><br>I asked the estate gamekeeper who was very pleased to give permission<br><br>In the past about 2002 I spent 4 days trying to locate landowners. The farmers are really on the edge(subsistence level) and most uncooperative about surveying. When I did find the land owner every year I had to write to him, contact Plantlife for a certificate of Public Liability send it off, let the land agent know when I was visiting. This is why I moved to the Path survey. I have only tried to survey squares and plots which have not involved seeking permission.<br><br>Long-standing permission since advent of Wildflower Count. No problem - personal contact.<br><br>almost all of the square is innacessible except for the right of way across it. The land is managed for a consortium of owners and is a highly contentious area which the council would like to release for building<br><br>all plots surveyed could be accessed within reasonable distance of a footpath<br><br>Granted with advice to mind the cattle: the field was empty when I went into it but just as I had identified the survey site the cattle found me and chased me off!

3.11 What were participants views of the survey?

Participants views of the survey were sought, Table 3. Accessibility, laying out square plots and entering data online are the key areas that participants identified as having had some difficulty with. Accessibility as an issue is even more pronounced when those self-selecting plots are factored out of the results. There is a clear need to strike a balance between directing surveyors to allocated plots and providing or equipping surveyors with simple and easy to interpret instructions with regard to what to do should they not be able to access those plots.

Table 3: Participant views of the survey

	Strongly agree or agree	Strongly disagree or disagree	Not applicable
**I was able to locate my square easily**	33	3	2
**I as able to lay out my square plot easily**	22	13	2
**I was confident in my ability to carry out the survey**	34	4	0
**I was confident in my ability to recognise habitats**	36	2	0
**There were no issues in accessing my plots**	20	18	0
**I as able to lay out my linear plots easily**	23	7	5
**I was able to record species listed easily**	27	6	4
**I was able to use the Domin scale easily**	30	2	5
**I was able to enter my data online easily**	15	12	8

The majority of participants posted additional questions with regard to how they had found the survey. A number of people commented on problems with locating and accessing woodland plots due to thick undergrowth. This reflects some of the experiences at encountered at the Juniper Hall residential and could be countered by encouraging surveys in woodland plots to be carried out earlier in the season. A number of people also identified river habitats as being difficult to survey due to overgrown vegetation.

There was also some frustration that the habitat descriptions did not match the grid reference locations and that access restricted surveyors being able to access target habitat. A couple of participants talked about GPS, one participant stating that it had become clear to him that the GPS on his iPhone was not that accurate. A number of people commented on the fact that setting up plots was a lengthy business – as inferred by responses to earlier questions.

Participants views on the guidance they were supplied with was also sought, Table 4 It is clear that more needs to be done with regard to the survey method guidance in particular and this was something reflected in additional comments supplied. It is suggested that a panel of volunteers is recruited to read through the guidance prior to printing and release. The lack of synchrosity in terminology between the guidance and the maps with regard to habitat descriptions was also flagged. There is also a need to improve the online guidance and the surveyor experience online.

"If you mean by 'online date guidance' (question 5 above) the guidance given on http://www.brc.ac.uk, I would say that there is insufficient guidance. I worked it out in the end (and it was easy) but I would have been glad of a brief description of the different pages that

needed filling. Again, on the data-entry, I was confused by the request for 'Description' on p.2. Also by the phrases in brackets: Plot number (used in plot name) & Grid Ref (used as plot name).”

Positioning the plots online when feeding back data was regarded as challenging by a number of participants and this clearly needs to be addressed. It is suggested that where possible plots are already marked on the map for a surveyor to record species against with surveyors only having to draw plots online when they have had to self select.

It is interesting to note that just under 30% of participants felt that there were insufficent species to record, supporting conclusions drawn above and that when inventory level surveyors are removed from this response pool the percentage of surveyors dissatisfied with the species list increases. The consultation survey mirrors these findings with circa 30% of surveyors recording at wildflower plot or indicator level reporting that there were insufficient species for them to record.

It is also interesting to note that participants generally felt that the habitat descriptions were helpful and understandable. Confusion about some habitats was flagged by Juniper Hall and has been raised by many volunteers participating in the wider survey this year. However it seems that those facing most difficulty with the habitats descriptions are recording at a pathway level rather than plot level.

Table 4: Participant views of guidance provided

	Strongly agree / agree	Strongly disagree/ disagree
**The habitats descriptions were helpful and understandable**	34	4
**There were sufficient species in my plot to record**	26	10
**The species lists were easy to understand**	32	5
**The online data guidance was easy to follow**	22	12
**The survey method guidance for my plot selection method was easy to follow**	17	19

3.12 What were volunteer experiences of recording additional attributes?

In general few of the volunteers had problems in recording the additional attributes. Steepness, management and vegetation height posed the greatest challenges. Six participants reported that these additional attributes were not easy to record. Only management type had one participant stating it was impossible to record. There is a contrast with the consultation survey where around 30 % of respondents (90) stated that they found most of the additional attributes listed not easy/ impossible. This can perhaps be explained that the pool of volunteers doing the field testing were perhaps more experienced. However this does have ramifications for the new NPMS and it is suggested that these additional attributes are reviewed and potentially fewer additional attributes recorded with the ones where there is greater uncertainty and probably accuracy of data removed.

3.13 Volunteer use of GPS

Participants were asked if they owned a GPS handset. Less than 24% of participants owned a GPS handset although just under 40% had GPS on their Smartphone / mobile. Over 90% of participates stated that they would not buy a GPS handset in order to participate in the survey. Of the people that owned a GPS handset or had access to it on their smart phone 37% used it to help them complete the survey.

It is reasonable to assume that the NPMS survey cannot rely on volunteers to own and use GPS handsets, also illustrated by responses to the consultation report. The methodology must therefore support people locate their plots as near as is possible to the plot location illustrated on the map

whilst providing language that dictates that proximity as opposed to exact location is within the tolerance levels of the survey.

3.14 What motivates volunteers to participate in the survey?

Participants were asked to select as many of the following comments as applied to them in order to gain a greater understanding of what motivates their participation (drivers) and therefore to inform key language with regards to promotion in 2015, Table 5. It is interesting to note that there is a difference between the self selected pool of field test volunteers and those who responded to the consultation survey. This is perhaps reflected by the number of people doing the wildflower pathway – enjoyment and learning being most frequently cited for this pool whereas contribution to plant conservation and the scientific community being more frequently cited by those involved in the field testing. It should be remembered that those doing the field testing had perhaps received more communication regarding the scientific nature of the survey.

Participants involved in the field testing were able to provide further comments and these reflected some concern or dissatisfaction that it will be important to consider as final amends are made to the NPMS and promotion starts. A number of participants stated that the survey had become more challenging with one stating that if you want to collect scientific data you should employ botanists. Perhaps the perceived increase in effort is not matched by reward. One participant felt that it was challenging to set up 10x10m plots and that there was no increase in species recorded, whilst another felt that the indicator level was frustrating as few species were recorded. However another participant stated “It was good to see different habitats and the larger squares produced more records” and another said “It was harder work going to different habitats but rewarding.”

A lot of people talked about time commitment. One participant suggested that communicating the increase in time needed in the first year to set up plots would be vital to new recruits whilst making it clear that this time investment would diminish in subsequent years. There were concerns and frustrations with regard to finding target habitat in the prescribed areas and a number of participants felt their enjoyment had been diminished through using the plot selection methods. One stated that recording abundance had meant more time was needed to complete the survey.

Table 5: Why do people participate in the survey

	% response field test	% responses consultation
I enjoy doing the survey	67	87
The survey gives me an opportunity to explore my local environment	70	65
The survey gives me an opportunity to meet new people	6	5
The survey gives me an opportunity to learn more about wild plants	81	79
The survey gives me an opportunity to contribute to the scientific community	86	68
The survey gives me an opportunity to help plant conservation	86	76

4.0 CONCLUSION

The field testing proved useful although participation was less than anticipated. Key emerging themes are the need to ensure there are clear protocols for self selecting plots should there not be enough accessible plots on the maps provided. In addition the species lists may require some revision.
