# Supplementary material for: The design, launch and assessment of a new volunteer-based plant monitoring scheme for the United Kingdom
Source: PLoS One. 2019 Apr 26;14(4):e0215891. doi: 10.1371/journal.pone.0215891 (PMC6485706; doi:10.1371/journal.pone.0215891)
Supplement: S4 File — (DOCX) [file pone.0215891.s006.docx]

# S4 File

**Selected volunteer feedback from field trials and online consultation**

*Survey guidance*

“I did find the plot guidance a bit complicated, which is partially why I did the [WFC] path survey only. Also, there weren’t enough different species to make it worthwhile doing plots.”

“I found the guidance good, but my lack of technical surveying experience probably let me down! I wanted to survey at the highest level I could, and hope that my lack of survey experience hasn't affected my results. I also found accurately recording my plots online a challenge! (Having no GPS was an issue here too).”

“I found it a bit overwhelming, but it got clearer once started.”

*Habitat identification*

“Some of the habitats we had before have disappeared from the list. What habitat is a roadside verge which is not a hedgerow or mainly grass? Why is there no mixed woodland habitat? It was not too difficult to identify a habitat for a plot but I had great difficulty knowing which plants I was looking for on different sections of the wildflower path which I surveyed last year.”

“Very difficult to find a habitat type matching country house parkland; neither pastureland nor urban quite fit. There used to be an improved grassland category which seemed to fit quite well but I could not find an equivalent; clearer definitions would help.”

“Identifying habitats has been difficult – my square has agricultural land, grazed grass and arable fields, conifer plantations and orchards, and these do not fit any of your listed habitats.”

“While the habitat descriptions were clear I had some difficulty in actually deciding what was what as the birch wood and pine wood tend to all flow together in differing proportions.”

“Habitat definitions are not comprehensive and too simplistic - fine in a perfect SSSI, NVC world but not in the real world.”

*Species identification*

“I didn't find many of the species listed for my habitat in the relevant list - this made me lack confidence.”

“I would like an ID guide for the expanded list of flowers (as in the previous survey). I don't feel confident in identifying everything on the expanded list. I found at least three plants on the survey I was unable to identify in spite of an hour studying my field guides and working through the Francis Rose keys.”

“‘Whenever I could I still used the previous guide but not all the species we are now asked to look for are in it. A new illustrated guide would be invaluable.”

“It would be good to have an image crib sheet available (as previous year) if required.”

“Because the species list was organised by habitat, I found it fiddly to decide whether a species was on the list or not. In the end we just identified everything we could and then I entered those that were on the list. It might be useful to provide downloadable mini guides where there are close matches to the listed species e.g. thistles, ragworts. Pictures of species in the booklet would be a great asset please.”

“A simple checklist of species which could just tick off rather than having to flick through whole book to work out if species in there.”

“Some of the new species added this year I had to look up online as I’d never heard of them and my ID book didn’t have it in it. I use the Wildflower Key by Rose but it didn’t have a few in it like skunk cabbage.”

*Land access*

“I have not tried to identify the owners of land in my squares partly because I did not find it easy in previous years for the Common Plant Survey or Wildflowers Count [WFC]. When I did manage to speak to the owner of some land he was very suspicious and did not really understand why anyone would want to look at plants on his land.”

“It is very hard to find out who owns the land and the only landowners whom I have spoken to have been suspicious about the reasons for someone wanting to have access to their land.”

“I don't have the confidence or the presence to approach landowners. Luckily my plots are on a public highway, a public footpath and an area of open access. I would not have done it if I'd had to approach a landowner myself, although if you were to pave the way for me I might consider it - and I am aware that this isn't helpful for the survey.”

“It is not always easy to find out who owns or manages a given piece of land. In the case of my plots, one is alongside a public bridleway, the other only a few metres from the bridleway and not fenced off, and I felt it was unnecessary to seek permission.”

*Volunteer motivations*

“Just wanted to say how much am enjoying this. Am slowly learning a bit each day about identifying plants.”

“I’ve really enjoyed doing it although it has been more time consuming than I expected, but the data site is easier to use than I anticipated.”

“‘Year by year the surveys are getting more objective and I hope more reliable. This latest scheme is by far the best and I hope that it encourages rather than discourages those of us who are at best interested amateurs.”

“I have done the first visit for four inventory plots in my square […] and enjoyed myself very much. As our farm sits on the cross between four squares would it be OK to do two other squares which I know have lots of interesting plants in them?”
