# Supplementary material for: The design, launch and assessment of a new volunteer-based plant monitoring scheme for the United Kingdom
Source: PLoS One. 2019 Apr 26;14(4):e0215891. doi: 10.1371/journal.pone.0215891 (PMC6485706; doi:10.1371/journal.pone.0215891)
Supplement: S5 File — (DOCX) [file pone.0215891.s007.docx]

# S5 File

## Global models for “assessing potential recording biases”

Analyses performed using car::Anova in R (Fox and Weisberg, 2011).

### GMEP vs NPMS indicator plots

Analysis of Deviance Table (Type II Wald chi-square tests)

Response variable: NPMS indicator species richness

|  | Chisq | Df | Pr(>Chisq) |
| --- | --- | --- | --- |
| Plot type | 52.30 | 1 | <0.001 |
| Habitat | 33.64 | 6 | <0.001 |
| Plot type:Habitat | 21.19 | 6 | 0.002 |

### GMEP vs NPMS inventory plots

Analysis of Deviance Table (Type II Wald chi-square tests)

Response variable: NPMS indicator species richness

|  | Chisq | Df | Pr(>Chisq) |
| --- | --- | --- | --- |
| Plot type | 0.23 | 1 | 0.63 |
| Habitat | 46.79 | 6 | <0.001 |
| Plot type:Habitat | 27.16 | 6 | <0.001 |

### References

John Fox and Sanford Weisberg (2011). An {R} Companion to Applied Regression, Second Edition. Thousand Oaks CA: Sage. URL: <http://socserv.socsci.mcmaster.ca/jfox/Books/Companion>
