# Supplementary material for: The design, launch and assessment of a new volunteer-based plant monitoring scheme for the United Kingdom
Source: PLoS One. 2019 Apr 26;14(4):e0215891. doi: 10.1371/journal.pone.0215891 (PMC6485706; doi:10.1371/journal.pone.0215891)
Supplement: S6 File — A brief overview of the approaches taken to promote the scheme, pre- and post-launch (2014–2016). (DOCX) [file pone.0215891.s008.docx]

# S6 File

**Promoting the NPMS.**

*Maximising recruitment*

The first stage in recruitment was to maximise the number of volunteers transitioning from Wildflowers Count to the NPMS (2014). Wildflowers Count volunteers were kept up to date with changes at every stage and were consulted regarding the development of the new scheme. As a result over 600 are now taking part in the NPMS.

The next stage was to promote the scheme as widely as possible using a national press release. The press release gained a known readership of 801,522 and led to a further nine radio interviews.

The promotion focus then turned to a local grassroots level and the NPMS flyer was used to contact all parish and community councils, local records centres, local natural history groups, University of the Third age groups, National Parks and AONBs. In order to reach different audiences outdoor user groups not already involved in biological recording were also contacted e.g. the British Mountaineering Council and the Ramblers. In a recent consultation of NPMS participants, 30% of respondents had an interest in the outdoors but had not previously been involved in survey work.

A plan for tackling remote areas was drawn up and this focused on developing professional development for national conservation agency and NGO staff, as well as on recruitment from local communities and outdoor enthusiasts.

Since the launch of the scheme on 1^st^ March 2015 there have been 2425 registrations on the NPMS website resulting in 1371 monads being allocated to volunteers (correct on 19^th^ October 2016). This is a good indication of the success of local level promotion.

*Importance of stakeholders*

The NPMS partnership recognises that there are a wide variety of stakeholders who have the potential to support the scheme. Many of these organisations may be able to “embed” our technique as a long term monitoring project across their land holdings, increasing uptake of monads and survey returns. A range of organisations have been approached by the NPMS including landowning conservation NGOs, protected area organisations and the biological recording community. Actions from stakeholders to date include promoting the scheme to staff and volunteers, taking ownership of monads that overlap with their land (facilitated by country-level workshops for Scotland and Wales), and providing expertise and financial support for training workshops. Workshops have also given these organisations the chance to express their ideas on how to improve uptake and to tell us how working alongside the NPMS could be beneficial to them. The two national workshops have been successful with lots of ideas and actions to be taken forward as the scheme progresses (please contact the corresponding author for copies of workshop reports).

**Table S5a**. A timeline of activities to maximise volunteer recruitment and participation etc.

| Month/Year | Support activities | Promotion and communication activities |
| --- | --- | --- |
| 2014 | | |
| November | - Production of survey pack materials - Training workshops organised |  |
| December | - Wildflowers Count volunteers contacted with their new NPMS square |  |
| 2015 | | |
| January |  | - Preparation of national press release |
| February | - Materials finalised and printed - Training plan online | - Email to volunteers regarding training |
| March |  | - National press release - Radio interviews |
| April | - Training - Email and telephone support |  |
| May | - Training - Email and telephone support |  |
| June | - Training - Email and telephone support |  |
| July | - Training - Email and telephone support |  |
| August | - Training - Email and telephone support | - Email to volunteers – participants questionnaire |
| September | - Training - Email and telephone support |  |
| October | - Data entry workshops | - Grassroots emails sent - Email reminder for data entry |
| November | - Training workshops organised | - Grassroots emails sent |
| December | - Mentor scheme developed | - Grassroots emails sent |
| 2016 | | |
| January |  | - Email to volunteers – can you take on an additional square |
| February | - NPMS mentors trained - Training plan online | - Email to volunteers regarding training |
| March | - Results fed back via newsletter | - Results newsletter sent to all contacts |
| April | - Training - Email and telephone support | - Email to volunteers - can you survey more plots – do you have coastal or montane habitats |
| May | - Training - Email and telephone support |  |
| June | - Training - Email and telephone support |  |
| July | - Training - Email and telephone support |  |
| August | - Training - Email and telephone support | - Email to volunteers – participants questionnaire |
| September |  |  |
| October | - Data entry support | - Grassroots emails sent - Email reminder for data entry |
